# Defining quality of healthcare in Dutch police custody: the development of a conceptual framework for monitoring care quality through a scoping review and expert consultations

**DOI:** 10.1186/s12889-026-27949-2

**Published:** 2026-07-02

**Authors:** Nienke D. Zinger, Vina V. N. Slev, Robert A. Verheij, Lotte Ramerman, Isabelle Bos, Dionne S. Kringos

**Affiliations:** 1 Netherlands Institute for Health Services Research, Nivel, Utrecht, The Netherlands; 2 Department of Forensic Medicine, Public Health Service, Amsterdam, The Netherlands; 3 Tilburg School of Social and Behavioral Sciences, Tilburg University, Tranzo, Tilburg, The Netherlands; 4 Health Care Institute Netherlands, Diemen, Netherlands; 5 Amsterdam UMC location University of Amsterdam, Public and Occupational Health, Meibergdreef 9, Amsterdam, The Netherlands; 6 Amsterdam Public Health, Quality of Care, Global Health, Amsterdam, The Netherlands

**Keywords:** Quality of care, Police custody, Incarceration

## Abstract

**Background:**

Individuals in police custody may require medical attention, yet little is known about the quality of care in police custody. Research is lacking on appropriate standards and indicators for measuring care quality. This study aimed to develop a conceptual framework to identify key aspects for measuring the quality of care in police custody in the Netherlands.

**Methods:**

A scoping review supplemented by expert consultations was conducted to gather information on health needs and quality of care in short-term police custody settings. Searches were performed across seven electronic databases (Embase, Medline, PsycInfo, CINAHL, Criminal Justice Abstracts, PiCarta, and Cochrane) for articles published in English or Dutch between 2008 and February 2023, and were supplemented with Dutch documents of medical guidelines, protocols, work instructions, and additional grey literature. An initial conceptual framework was developed from the literature review and author expertise, which was subsequently discussed and validated by field experts through two expert consultation group meetings.

**Results:**

This study included 69 scientific articles, 17 medical guidelines, protocols and work instructions, and 12 reports, alongside consultation with 27 experts. The resulting conceptual framework applies a structure-process-outcome model to evaluate the care quality in police custody, organized into 14 domains. At the structure level, it includes the scope and nature of care demand, (healthcare) staff, the legal framework, quality assurance, infrastructure and (medical) resources, and funding. The process level covers triage and access to healthcare, detection of care needs, continuity of healthcare information, coordination of care, general healthcare provision, and healthcare provision for common needs. The outcome level addresses effectiveness and health outcomes, and satisfaction. These domains are further classified into 48 subdomains. There were no existing indicators identified in the literature to populate the framework.

**Conclusions:**

The conceptual framework outlines key domains for measuring quality of care in Dutch police custody. Future research will be necessary to populate the framework with indicators to monitor quality of care and support improvement efforts tailored to the needs of specific stakeholders. The operationalization and implementation of the framework will be vital for improving the care provided within police custody.

**Supplementary Information:**

The online version contains supplementary material available at 10.1186/s12889-026-27949-2.

## Introduction

The right to health is recognized in several human rights instruments and applies equally to persons who are detained [1]. In accordance with the principle of equivalence, states are required to ensure that primary care services for detained persons are delivered at standards equivalent to those applicable to the wider community [2–4]. Nevertheless, detained individuals face restricted access to providers and rely on detention guards for their access to care, exposing them to a vulnerable situation regarding health and well-being in custody. In addition, the detained population disproportionately consists of individuals with greater health disparities compared to the non-detained [5, 6]. Inadequate assessment and failure or denial of access to healthcare services in custody can result in health complications or even death, if serious health conditions are improperly addressed [1].

Within the Dutch justice system, police custody is the initial stage of detention. Police custody can generally be defined as short-term detention, typically lasting up to a few days, although variations exist between countries [7]. In the Netherlands, a detainee can be held in police custody for a maximum of 6 days before either being released or transferred to another justice facility [8, 9]. The medical care is provided by physicians (forensic physicians, general practitioners, and non-specialist physicians) and (forensic) nurses, who attend police custody facilities on an on-call basis, often covering multiple facilities and sometimes simultaneously fulfilling clinical duties outside the custody setting. The healthcare provision is contracted by the police and organized regionally, remunerated through a fee-for-service system, and in some cases supplemented by an additional lump-sum arrangement. Accordingly, the healthcare services are publicly funded, with the exception of prescribed medications, which are sought to be handled via the detainees’ health insurance.

Information derived from quality indicators can guide quality improvement initiatives and the implementation of evidence-based practices in learning health systems [10, 11]. However, knowledge is limited regarding the quality of care that is provided in Dutch police custody. Research on appropriate standards and indicators to measure quality of care is currently lacking and no consensus has been reached yet on how to define care quality in this specific context. For prison health systems, international efforts have been made to develop performance measures [12–14]. However, measures designed for such longer-term incarceration settings cannot simply be applied to short-term custody settings.

Police custody environments are characterized by limited available medical information, rapid turnover of detainees, and constrained medical infrastructure. Healthcare in this setting is primarily focused on acute conditions and immediate care needs that cannot be deferred until release or transfer to a longer-term detention setting [15]. In contrast, prison settings are generally oriented toward longer-term care and continuity of treatment over extended periods of incarceration. Police custody also differs from other acute and primary care settings in that the police have a duty to facilitate access to healthcare professionals when necessary. Hence, there is a need for instruments that provide insight into the quality of care specifically within police custody settings.

To develop quality indicators that capture the full scope of care in this context, a clear understanding of the components of good quality care in this setting is required. Therefore, this study aims to develop a conceptual framework that captures all relevant aspects that are essential for measuring quality of care for people detained in Dutch police custody. To identify critical aspects of healthcare delivery and quality assessment, this study examines the healthcare-related activities within police custody. It also aims to understand prevalent health issues and specific detainee requirements crucial to tailor healthcare services, ensuring the wellbeing and adequate medical care of this population during police detention. Additionally, this study seeks to identify key domains pertinent to measuring care quality that are essential for effective quality assessment and improvement efforts within these settings. This also includes the understanding of the organizational structure and processes governing the healthcare provision in police custody. Finally, the study examines whether indicators already exist that are designed to assess quality of care in short-term custody settings and the type of data they use.

These steps serve as the foundation for the development of a conceptual framework outlining the key domains of quality of care for people detained in short-term police custody. The conceptual framework could act as a structured guide for understanding and enhancing quality of care within Dutch police custody settings. The conceptual framework that delineates structural elements alongside care processes and outcomes, will help to prioritize areas for measurement and intervention, facilitating systematic evaluation, measurement, and improvement of health system structures and care processes.

## Methods

This study was conducted in three sequential steps. First, a scoping review was conducted. As literature on quality of care in Dutch police custody is limited, international scientific literature was included to gather more information on health needs and quality of care in police custody. To incorporate the Dutch context, Dutch medical guidelines, protocols, work instructions, and grey literature were analyzed. Second, the authors synthesized the findings from the review to construct a conceptual framework encompassing key aspects of quality of care for individuals in police custody. Third, expert consultation meetings were convened to refine, complement, and validate the conceptual framework.

### Scoping review

#### Study design

The scoping review involved peer-reviewed original research articles, medical guidance documents (e.g. medical guidelines, protocols, and work instructions), and grey literature (e.g. policy documents and reports). The methodology followed Arksey and O’Malley’s framework [16] to synthesize the existing evidence on the provision and quality of care for people detained in short-term police custody settings and included expert consultations. The reporting adhered to the Preferred Reporting Items for Systematic reviews and Meta-Analyses extension for Scoping Reviews (PRISMA-ScR) checklist [17].

#### Inclusion and exclusion criteria

Publications written in English or Dutch were included if they addressed health needs of police custody detainees, healthcare provision, or information or recommendations about quality of care in short-term police custody settings. Publications discussing care-related topics (e.g. sharing of information) at detainee transfers from the community into police custody, as well as from police custody to the community or another detention facility, were also included. Publications on the feasibility or effectiveness of interventions within the police custody setting were eligible for inclusion when it discussed recommendations on how to measure or improve quality of care. Publications about death in custody that discussed health-related causes of death were included, although the search terms were not specifically targeted at death in police custody studies.

Publications were excluded if it was unclear whether the setting pertained to short-term police custody. This was due to differences in terminology, unspecified maximum length of stay, or ambiguity regarding whether it addressed the first detention facility after arrest. Publications related to asylum detention settings and those that primarily focused on human rights issues rather than on healthcare provision itself, were also excluded. Additionally, opinion articles, book chapters, and conference and dissertation abstracts were excluded.

#### Search strategy

Searches were conducted on seven online databases: Embase, Medline, PsycInfo (all via the Ovid platform), CINAHL, Criminal Justice Abstracts (all via the Ebsco platform), Cochrane, and PiCarta (based on Dutch Central Catalog and bibliographic records of scientific journals available in Dutch libraries). The database searches were restricted to literature published between January 2008 and February 2023. Search strategies were developed combining two types of strings. An example search is included in Supplementary file 1. The first string included concepts related to healthcare or quality of care, including ‘health needs’, ‘medical care’, ‘medical examination’, ‘quality indicator’ and ‘quality of care’. The search terms for quality of care measurement were informed by the findings of Dudley et al. [18]. The second string targeted the short-term police custody setting and incorporated a range of internationally used terms, following Wardrop et al. [19], e.g. ‘custody’, ‘detained’, ‘arrested’, and ‘watch house’.

Relevant Dutch medical guidance documents and grey literature were identified via websites of organizations involved in healthcare delivery in police custody (e.g. Dutch police and police custody healthcare providers), as well as through searches conducted in Google using search terms comparable to those applied in the international scientific literature search, combined with “Netherlands”. Additionally, members of the advisory committee of this study were asked to share relevant documents with the research team.

#### Publication selection

After duplicate removal, two authors independently screened 61% of the abstracts of all retrieved publications. The remaining abstracts were reviewed by one author (NZ). Publications that raised doubts regarding inclusion were marked for full-text review. Subsequently, 53% of the full-texts were independently screened by two authors. Discrepancy meetings were held during the review process to compare results and ensure consistency in applying inclusion criteria. Publications screened by one author (NZ) were reviewed by a second author (IB) in case of doubt. Publications on jail settings were initially included for full-text review but excluded if the study population involved detainees with stays exceeding one week, as this fell outside the study’s focus on short-term custody settings. The PRISMA-ScR flow diagram was used to describe the screening procedure. The medical guidance documents and grey literature were screened for relevance based on their summaries and headings and were included if they contained information on care for individuals detained in short-term police custody settings. One additional relevant scientific publication was identified through the grey literature search and added to the final set of included studies. The grey literature search was stopped once data saturation was reached in combination with the already included scientific publications. EndNote and Rayyan were used to manage the screening process, and Excel was used to randomly assign publications to a second reviewer.

#### Data extraction and synthesis

Data extraction was performed by one author (NZ) using a structured data collection form. Initially, a summary of each article was created, encompassing author(s) names, publication year, study setting, data source, and country. Subsequently, specific details were extracted regarding the activities of police custody healthcare providers, recorded health issues and needs of detainees, recommendations regarding aspects of quality of care, and – if available—defined quality indicators. The same type of information was also extracted from the medical guidance documents and grey literature. Finally, the extracted information regarding quality of care was categorized by the authors into overarching domains and subdomains.

### Conceptual framework development and expert consultations

#### Preliminary conceptual framework

A preliminary conceptual framework for measuring quality of care in police custody was developed by categorizing identified key aspects into domains and subdomains, drawing on the scoping review outcomes and leveraging the authors’ (RV, DK) expertise in health systems performance assessment and quality of care. The identified domains and subdomains were subsequently organized into structures, processes, and outcomes to discern the conditions and arrangements that should be in place, the occurrence of health problems and actual delivery of healthcare processes, and the resulting outcomes [20].

#### Expert consultation group meetings

Two separate online expert group consultation meetings were convened to refine, complement, and validate the conceptual framework. Participants were recruited through outreach to organizations involved in police custody healthcare (e.g. healthcare providers, police, pharmacies, mental healthcare providers, educational institutions), snowball sampling, and the researchers’ professional network. Participants received the draft conceptual framework one week prior to the meeting. During the meetings, the framework was explained and subsequently discussed. Participants were asked to review the framework and provide suggestions for refinement. The meetings were recorded, transcribed, and summarized.

Both groups represented a broad range of perspectives and extensive experience in healthcare delivery in Dutch police custody (Table 1). The first expert group consisted of professionals directly engaged in the provision of care for police custody detainees. Their two-hour meeting began with a plenary session where the first draft of the conceptual framework was further explained. Thereafter, four breakout sessions were conducted, each focusing on a specific topic: 1) detainee arrival and health needs assessment, 2) providing healthcare, 3) individuals with mental health issues or requiring medication, and 4) transfer from police custody to another (care) setting. The experts were allocated to the groups according to their respective area of expertise. Each breakout session was led by one of the authors. The meeting concluded with a plenary session in which each group shared its conclusions and the draft conceptual framework was discussed once more. Divergent views among experts were handled through consensus discussion.

**Table 1:** Overview of experts who participated in the consultation meetings of the conceptual framework (*n* = 27)

Participants of the first expert consultation group meeting	Participants of the second expert consultation group meeting
• Physicians providing healthcare in police custody (*n* = 6) • Forensic nurses providing healthcare in police custody (*n* = 1) • Police custody officer (*n* = 1) • Pharmacist delivering medication to a police station (*n* = 1) • Physician (*n* = 1) and nurse (*n* = 1) providing healthcare in custodial facilities • Physician of Dutch Street Doctors Group (*n* = 1) • Scientists specialized in forensic medicine (*n* = 2) • Educator of forensic medicine (*n* = 1) • Netherlands Public Prosecution Service (Medical Affairs Expertise Center) (*n* = 1)	• Healthcare organization managers and care coordinators (*n* = 4) • Dutch Forensic Medical Society representative (*n* = 1) • Dutch Nurse Association representative, section forensic nurses (*n* = 1) • Police custody officers representative (*n* = 1) • Dutch Custodial Institutions Agency (medical advisor and forensic physician) (*n* = 1) • Advocacy of detainees (Dutch independent advocacy group for (former) detainees and their families) (*n* = 1)* • People with lived experience of police detention (*n* = 2)

^*^Could not attend the meeting and was consulted individually at a later time

Following the first meeting, the conceptual framework was revised and circulated to the second expert group. This group included participants at the managerial level and representatives of professional associations involved in the provision of care for detainees in police custody. Final adjustments were made during this meeting, resulting in the final version of the framework. To ensure that all critical topics important for detainees were included, two individuals with lived experience of detention in police custody met individually with the authors to share their views and experiences regarding healthcare provision in this setting.

The iterative process of redrafting and discussion led to evolving insights into the interpretation of the literature. Consequently, several subdomains proposed during the consultation meetings were later also identified in the literature.

## Results

### Findings from the scoping review

#### Characteristics of the publications

**Fig. 1:**
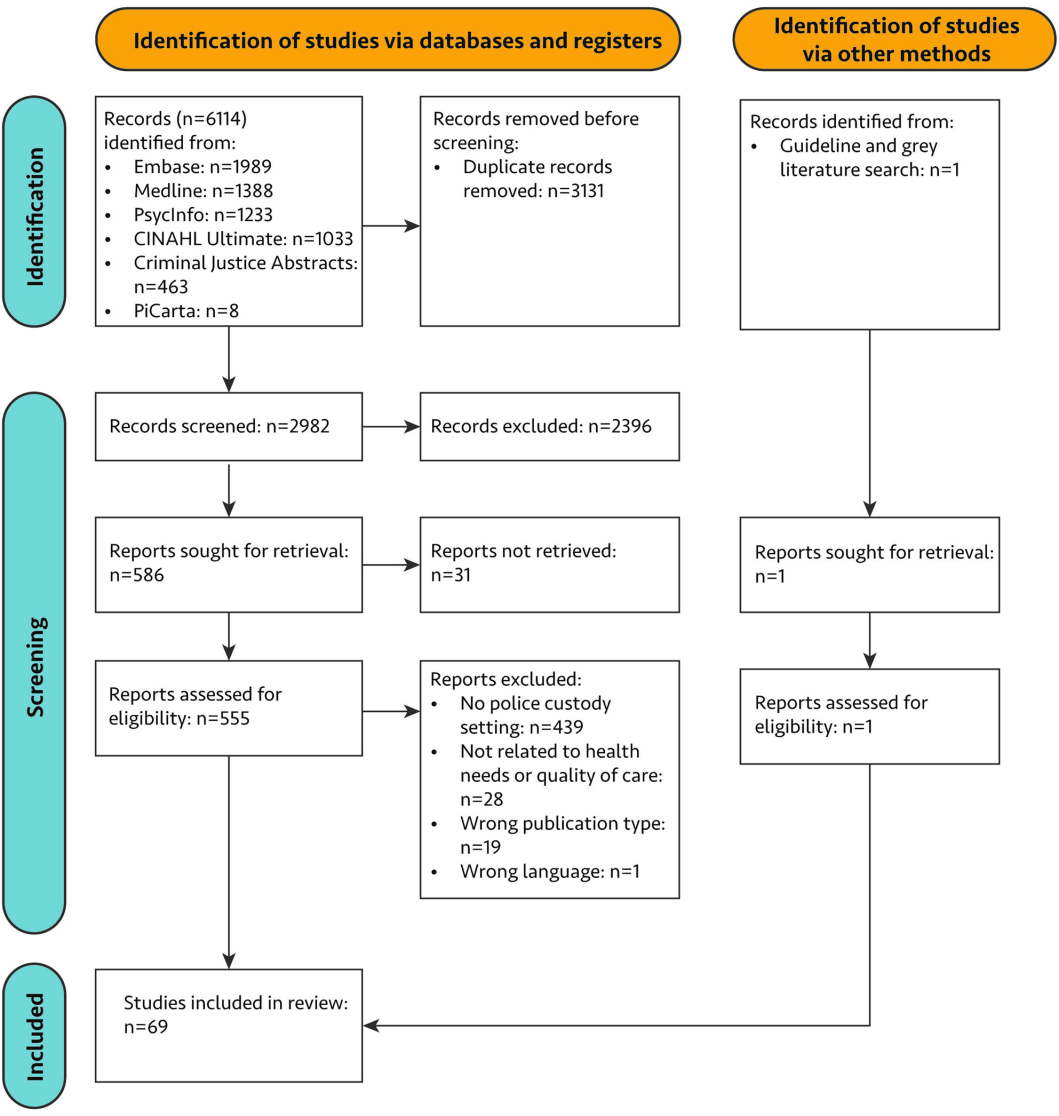
PRISMA flow diagram of peer-reviewed publications

After removal of duplicates, titles and abstracts of 2982 publications were screened, and 555 publications were reviewed in full-text (Fig. 1). Following full-text review, 487 publications were excluded, including 439 that did not specifically address police custody, but instead focused on longer-term jail settings or were unclear regarding the study setting. With one additional peer-reviewed publication identified through the grey literature search, a total of 69 scientific publications were included. Most of these studies focused on the health status and treatment of detainees in police custody. None addressed the development or measurement of quality indicators for police custody settings. However, 52 studies provided recommendations for maintaining or improving quality of care. The majority of studies were from the United Kingdom (*n* = 24), France (*n* = 16) and the Netherlands (*n* = 10) (see Supplementary file 2 for details on the included publications). The searches for medical guidance documents and grey literature yielded 17 medical guidelines, protocols, and work instructions, and 12 reports.

#### Data sources

Various data sources were employed in the included publications to report on the health problems of detainees during custody. Frequently used data sources included data collected from medical examination records and questionnaires completed by healthcare providers during or after consultations. These data contained information on verified health conditions, as well as self-reported information by the detainee [21–43]. Other data sources included police custody records [31, 40, 44, 45], interviews with detainees carried out by researchers or psychiatrists [21, 44, 46–48], registers of psychiatric and mental health services [21, 49, 50], a questionnaire for detainees [34, 39, 51], and an urine specimen analysis [52].

#### Healthcare-related activities within police custody

Three studies reported the overall percentage of detainees for whom a healthcare provider was contacted during the police custody period, which was the case for around a quarter to almost half of the detention episodes [21, 22, 40]. The proportion of the medical examinations requested by the detainees themselves varied across studies, with the highest value reported being 40% of the examined individuals [23, 25, 27, 33, 38, 42]. In one study, 3% of the examined detainees were found to have ongoing medical problems, even though they did not report any health issues [26]. According to six studies, between one to seven percent of the examined detainees were transferred to a hospital for further treatment [22, 24, 25, 36, 37, 45].

Healthcare-related activities in police custody included several types of assessments, encompassing assessments of physical health problems [28, 31, 37], mental health problems [28–31, 34], and for fitness for custody [23–25, 27–29, 31–34, 37, 38, 40, 42]. Activities related to treatment provision [23, 25, 27, 31–33] included the prescription and administration of medication in general [22, 24, 26, 27, 31, 33, 37, 38, 42, 43], as well as substitution medication of opiate, narcotic or alcohol abuse [35], and methadone prescription [21, 37]. Full details about the reported healthcare-related activities in police custody are included in Supplementary file 3.

#### Health problems of detainees in police custody

The studies reported a variety of health problems (see Supplementary file 4 for details). Mental and psychiatric health problems in general were reported for 22% to 39% of the detained population, and between 9 and 50% of those who were examined by a healthcare provider [21, 28, 29, 31, 35, 43, 46, 49]. The prevalence of somatic disorders reported in the medical examination records ranged from 7% of the examined younger detainees to 77% of those over 60 years of age [22–25]. Medication use at time of arrest was reported for a fifth to around half of the detained population, and 67% for those aged 50 and over [28, 31, 44, 47]. Pregnancy was reported in up to 7% of the examined female detainees during custody [38, 40, 47].

A study by Bennett and Holloway found that 72% and 66% of the detained population tested positive for drugs for those aged 17–24 and over 25, respectively, who agreed to participate in an urine specimen analysis [52]. Drug-related medical problems were reported by Buster et al. in 8% of the examined population [21]. Regarding alcohol use, McKinnon et al. estimated that 15% of detainees aged 18–49 and 26% of those aged 50 or older were at risk of alcohol withdrawal [44]. Sturgiss and Parekh reported that 18% of the examined population was intoxicated with drugs or alcohol at the time of arrest [37].

Self-reported health concerns of detainees while in custody were described by three studies. In a study by Brooker et al. health concerns were expressed by 22% of the detainees [51]. Of the participants in a study by Baksheev et al., 11% reported access to healthcare as a major concern during police custody [48]. The most frequently reported specific concerns were related to medication (8%), drug (6%) or alcohol (4%) problems [51]. Concerns about self-harm or suicide while in custody were reported for less than 1% to 18% of the detainees [46, 48, 51].

#### Death and self-harm behavior in police custody

Six studies reported on death in police custody [53–58]. Unnatural causes of death included violence (e.g. homicides, deaths as a result of legal interventions), suicide, drug and/or alcohol intoxications, poisoning from medical and illegal drugs, alcohol withdrawal delirium, craniocerebral trauma, and traumatic injuries [53, 54, 56–58]. Natural causes of death were attributed to a variety of issues, among them myocardial infarction, infectious diseases, and pneumonia [53–57]. Regarding self-harm behavior in custody, Cummins et al. found that two-thirds of self-harm cases were associated with recorded conditions of being ‘drunk’ or ‘under the influence of alcohol or drugs’ [59].

#### Recommendations about quality of care

Table 2 summarizes the domains and subdomains derived from the scientific literature, medical guidance documents, and the grey literature. Frequently discussed domains in the peer-reviewed literature covered detainees’ access to care [19, 51, 57, 58, 60–64], continuity of (healthcare) information [35, 36, 45, 57, 65–70], coordination of care [23, 29, 45, 46, 49, 52, 64, 67–69, 71–80], and staff capacity and education [31, 56, 57, 59–61, 65, 67, 71, 81–84]. Medical guidance documents often focused on detection of care needs [85–88], including signaling and continuous monitoring, and healthcare provision for specific health problems or risk factors [86, 88–94]. The grey literature provided additional insights into the coordination of transitions to other justice facilities and healthcare services after release [95–98].

Across all types of literature, recommendations were made regarding the quality system of care provision in police custody [31, 45, 58, 65, 67, 83, 86–88, 90, 95, 97, 98, 101, 102, 108–111]. The significance of strong registration systems was emphasized to ensure accurate record-keeping, effective information transfer, patient safety, and thorough quality performance reviews. Additionally, clear and up-to-date multidisciplinary agreements were recommended to streamline efficient care provision, particularly given the many parties involved in the care for police custody detainees.

All recommendations extracted from the scientific literature search are listed in Supplementary file 5.

**Table 2:** Domains and subdomains derived from recommendations about quality of care in short-term police custody settings

Domain	Subdomain	Description	Scientific literature	Medical guidelines, work instructions, and protocols	Grey literature
Access to care	Medical assessment	Adequate assessment and identification of physical and mental health issues by police officers and seeking medical attention where required	[19, 57, 58, 60, 63]	[86–88]	[99]
	Medication	Access to required medication during custody	[64]		
	Referral	Access to specialized healthcare services (e.g. hospitals) if the police custody setting is not equipped to deliver the necessary care for the detainee	[57]	[88]	
	Timeliness	Appropriate length of time to access healthcare	[19, 51, 58, 61, 62]	[87]	
Continuity of information	Availability of information	Availability of information from previous detention episodes or other organizations within the healthcare chain (e.g. mental healthcare providers, hospitals)	[35, 36, 45, 65–69]	[86, 88]	
	Communication	Adequate, clear and comprehensible communication between the healthcare provider and the police	[57, 67, 70]	[86, 88]	[95]
Coordination of care	Collaboration	Collaboration with other healthcare services (e.g. mental healthcare providers, general practitioners, addiction treatment services) for brief interventions or onward referral for unmet health needs	[23, 29, 45, 46, 49, 52, 67, 68, 71–76, 78–80]	[86]	
	Coordination of transition	Coordination of transition from police custody to prison healthcare providers or other health services	[64, 69, 77]	[86, 88, 93]	[95–98]
Detection of care needs	Detection of care needs of detainee	Identification of healthcare needs of a detainee (e.g. diagnosis, treatment, medication, observation)	[83, 100]	[85–87]	[101]
	Monitoring	Supervision and structural monitoring by the police to detect deterioration of health conditions and care needs. Including healthcare needs developed after the initial health risk screening	[19, 56, 57, 61]	[85, 87, 88]	[102]
Effectiveness	Health outcomes	Detainee’s health outcomes after the police custody detention period	[19, 103]		
Funding	Funding of healthcare provision and treatment	Funding system through which the healthcare provision and treatment is financed		[86]	
Healthcare provision	Care for urgent needs	Provision of necessary medical care to detainees, including the continuity of care regarding any ongoing treatment or medication	[24, 83]	[86, 89]	
	Medication	Provision of medication, including medication prescription and dispensing	[61]	[86, 88]	[98, 104]
Healthcare provision for specific populations or frequently encountered health problem	Alcohol withdrawal	Care for detainees at risk for alcohol withdrawal symptoms	[25, 105]		
	Drug withdrawal	Care for detainees at risk for drug withdrawal symptoms	[41, 58, 66]	[90–93]	[96]
	Intellectual disability	Care for detainees who have an intellectual or learning disability		[88]	
	Mental/psychiatric health problems	Care for detainees experiencing mental or psychiatric health issues	[48]	[86]	
	Minors	Care for detainees younger than 18 years	[32]	[88, 94]	[106]
	Older individuals	Care for detainees of older age	[25, 44]		
	Pregnancy	Care for pregnant detainees and addressing reproductive health needs	[38, 67, 107]		
	Self-harm/suicidal behavior	Care for detainees with a threat of self-harm or suicidal behavior		[86]	
Infrastructure and (medical) resources	Cell provisions	Provisions within police cells for a certain level of living conditions. E.g., the availability of sanitary towels for female arrestees, beds with properties that do not carry the risk of fatal falls, and restriction of items useful to inflict self-harm	[25, 38, 56, 57, 61]		
	Clinical diet	Availability of meals suitable for certain medical conditions (e.g. for diabetes management)	[65, 67]	[86]	
	(Medical) equipment	Availability of equipment for providing healthcare within police custody	[61, 65]	[86]	
	Interpreter	Availability of facilities that ensure the interaction between detainees and healthcare providers or police custody officers is conducted in a language and terms both parties can understand	[61]		
	Medication	Availability of infrastructure for the prescription, storage, and dispensing of medication		[86, 88]	[101]
Legal	Medical confidentiality	The balance between keeping medical confidentiality and the disclosure of information to the police in order to ensure proper monitoring	[61, 77]	[86]	[95, 102, 104]
Quality systems	Handling of complaints and incidents	Procedures for handling and learning from complaints and incidents		[88]	[95, 108]
	Medication safety	Existence of quality systems around medication. E.g. checking current prescriptions, expiry date of medication brought from home, and administration to the right detainee	[65, 67]		[95, 97, 102]
	Multidisciplinary agreements	The establishment of multidisciplinary work procedures and agreements for all involved parties	[31]	[86]	[97, 109]
	Performance reviews	Procedures around reviewing quality standards, performance, complaints and incidents	[31, 45, 83, 110]		[111]
	Registration	Registration systems about the care provision for the police and healthcare providers	[58, 110]	[86–88, 90]	[98, 101, 111]
Satisfaction	Satisfaction and complaints	Satisfaction and complaints of detainees, healthcare providers and police, including issues and satisfaction related to the dignity of their treatment by the police, as well as healthcare providers	[64]		[111]
Scope and nature of the care demand	Availability of information about the care demand	Availability of information about the scope and nature of the care demand of detainees in police custody	[103]		
Staff	Education and training	Education and training of police and healthcare professionals involved in the care provision for detainees	[31, 56, 57, 60, 61, 65, 67, 71, 81, 83, 84]	[61, 86]	[99, 104]
	Skill mix and capacity	Availability of skill-mix among staff, interprofessional collaboration, and capacity of the available workforce	[31, 57, 59, 60, 82]		

### Findings from the expert consultation meetings and development of the conceptual framework

#### First draft of the conceptual framework

Initially, five structure domains were identified to describe the key aspects of quality of care in police custody. The domains: scope and nature of the care demand, infrastructure and (medical) resources, (healthcare) staff, and quality assurance, were identified through the literature review. One domain was added by the authors, recognizing the crucial role of funding for both healthcare provision and treatment in determining the overall effectiveness and sustainability of care systems, as well as its impact on the availability of quality healthcare services. For the processes related to quality of care in police custody, six domains were identified through the literature review: access to care, detection of care needs, continuity of healthcare information, coordination of care, and two domains on healthcare provision. The healthcare provision process is described in two domains: one to describe general processes around healthcare provision (e.g. the prescription and dispensing of medication), and one to describe specific healthcare provision processes for frequently seen health needs. With respect to outcomes, one domain on health outcomes named ‘effectiveness and health’ was identified through the literature review. An additional outcome domain on satisfaction was added by the authors to ensure a comprehensive assessment of care quality, as satisfaction is crucial for evaluating the overall effectiveness of healthcare delivery and identifying areas for improvement.

#### Expert consultation meetings: Conceptual framework for measuring quality of care in police custody

The experts in the first consultation group recommended adding a structural domain focused on the legal framework governing care in police custody. The corresponding subdomains, access rights to medical records and medical confidentially, were suggested by the second expert consultation group. The grey literature offered further recommendations on balancing medical confidentiality with the need for adequate healthcare provision [95, 102, 104].

Additionally, the first consulted group of experts suggested to place more emphasis on addressing complaints. Consequently, subdomains about complaint handling were added to the structure domain of quality assurance (availability of a system for handling complaints), the process domain of general healthcare provision (the actual handling of complaints), and to the outcome domain of satisfaction. The experts also emphasized the importance of assessment and triage to ensure timely and good healthcare accessibility, which was added to the domain on access to care.

**Fig. 2:**
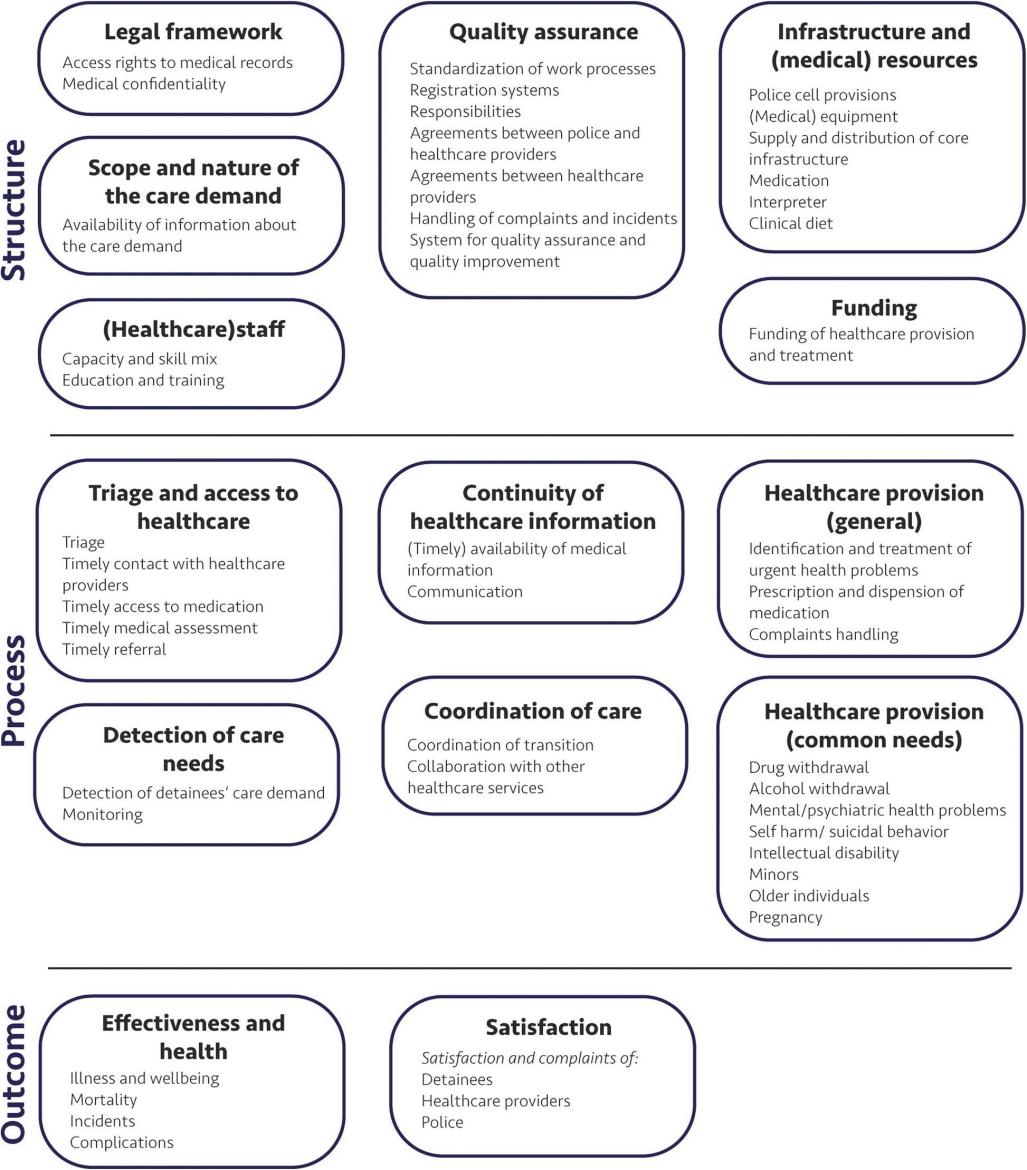
Conceptual framework for measuring and improving quality of care in short-term police custody settings

The second expert consultation meeting resulted in the addition of several subdomains. A structural subdomain was added to address how the number and distribution of core infrastructure elements can impact quality of care, such as travel times between police stations or the availability of observation cells. In terms of finances, the experts noted that the type of financing for healthcare provision affects quality. Since in the Netherlands healthcare provision (e.g. assessments) in police custody is financed differently from treatment provision (e.g. medication dispensing), an additional financial subdomain was included in the framework. The refined and validated conceptual framework is shown in Fig. 2.

## Discussion

This study aimed to address the gap in research on healthcare quality in police custody settings by developing a comprehensive conceptual framework. Despite extensive literature and expert input, a noticeable gap was shown in quality indicators specifically tailored to the unique challenges of healthcare in short-term police custody settings, including the role of the police as an intermediary in access to care, detention stays of a few days at most, and a population characterized by a high burden of physical and mental health conditions.

The developed conceptual framework identifies 14 key domains essential for evaluating healthcare quality in this context, encompassing preconditions (structures), process, and outcome aspects. The conceptual framework integrates structural elements (e.g. staff capacity, registration systems, and funding), process domains (e.g. access to care, continuity of information, and coordination of care), and outcome domains (e.g. health outcomes, and satisfaction). This holistic approach ensures a comprehensive evaluation of both operational and systemic factors, facilitating targeted improvements in quality of care for police custody detainees.

Detailed information about health problems, needs, and concerns of detainees in police custody is needed to organize healthcare provision towards meeting the urgent care needs. The literature revealed that healthcare providers were contacted for approximately a tenth to half of the detained population for a variety of issues including somatic, mental health, and substance use disorders. The healthcare-related activities included the execution of a variety of health assessments, treatment provision, and the provision of medication. Studies reporting on detainees’ self-reported health concerns found that up to a fifth of the detainees expressed a health concern while in police custody.

Most studies described the detainees’ health issues based on healthcare provider observations, and some used detainees’ self-reported concerns. Both measures have their own factors influencing the reliability and validity of the data [112–116]. Notably, studies often excluded detainees with language barriers, potentially underreporting the health needs of vulnerable groups such as undocumented migrants. This limitation underscores the importance of inclusive research that accurately reflects the diverse health needs and concerns within police custody.

The conceptual framework’s emphasis on assessment, triage, and timely access to care aligns with the need to address immediate health issues efficiently, potentially reducing the need for external hospital transfers and optimizing resource use [45]. A well-coordinated transition from police custody to other healthcare providers can also enhance the efficient use of resources, for example by sharing information on completed assessments or ensuring that any medication allocated to the detainees are transported with them.

Good collaboration practices and health information exchange are key to facilitate adequate healthcare provision within and outside police custody. Relevant medical information helps healthcare providers in police custody to make informed decisions about necessary care and ensuring appropriate medication prescriptions. A lack of access to health data from police detention by community healthcare providers or providers in other justice facilities disrupts continuity of care [117, 118]. However, the exchange of health information can be complicated by several factors, including medical confidentiality issues, poor interoperability between health information systems, detainees’ possible reluctance to consent to sharing information about their time in detention, and a lack of registration with a general practitioner [22, 119].

Whereas the current Dutch healthcare provision in police custody is limited to providing care that cannot be postponed, an expansion of the care provision may offer an opportunity to improve public health and reduce health inequalities by serving as a gateway to the community healthcare system [6, 120]. Existing initiatives demonstrate that collaboration between police and health services can facilitate improved health outcomes for detainees and support community health [50, 121, 122]. Early identification and management of health issues during custody, including brief interventions for addiction and preventive measures for communicable diseases, can help mitigate long-term health consequences for detainees and the broader community [26, 123]. Ultimately, incarcerated individuals will return to the community, potentially carrying untreated conditions or infectious diseases that may threaten public health and increase the community’s disease burden [2, 117].

The literature review yielded few publications on the quality of care in police custody. No publications were found that provide an overview of care quality domains or performance measures specific to this context. Whereas recommendations about quality of care in the peer-reviewed literature focused on themes such as access to care, continuity of information, coordination of care, and capacity and skills of staff, the medical guidance documents and grey literature emphasized aspects of the healthcare provision itself. The conceptual framework can assist in pinpointing areas where peer-reviewed scientific literature or medical guidance documents are lacking, which may impede evidence-based improvement initiatives.

### Implications for practice

The conceptual framework developed in this study can serve as a structured guide for understanding and enhancing the quality of care within the police custody setting. The framework helps prioritize structure, process, and outcome areas for measurement and intervention, and can guide the development of actionable quality indicators tailored to the unique context of care delivery in police custody. When developing actionable indicators, their fitness for purpose and fitness for use should be considered [124]. Using routine healthcare data or police custody records to measure quality indicators limits the registration burden for the professionals involved. Although more time-intensive, a survey of detainees’ self-reported health status could provide valuable additional information on quality of care, such as frequently reported health concerns as well as detainee satisfaction.

Care should be taken during the development phase of quality indicators to ensure they allow healthcare providers to make decisions based on individual and medically assessable factors, so that the medical aspects of police custody do not become over-regulated [57]. Quality indicators should be designed in a manner that ensures the information derived balances the healthcare providers’ professional autonomy while serving as a foundation for informed discussions regarding actions to maintain or improve quality of care [125]. Collecting quality indicator outcomes assists in identifying practices in which safe, timely and effective care is delivered [126]. If the knowledge derived from indicators is effectively translated into practice, the development and use of quality indicators can make a significant contribution to a learning healthcare system in police custody. To operationalize the conceptual framework into quality indicators, involvement of representatives from all relevant stakeholders such as police, healthcare providers, and policy makers is recommended to ensure an integrated set of indicators and foster a shared sense of ownership. Furthermore, implementation of indicators in practice requires consideration of how their use may influence practice, including potential positive and negative effects, and calls for agreements on continuous monitoring of indicators and on evaluating their use and impact, and decisions regarding any consequences.

To our knowledge, this is the first conceptual framework that outlines all key domains essential for describing quality of care in police custody. Police custody settings in other countries could also benefit from this framework to develop quality indicators. However, when applying this Dutch context-based framework internationally, contextual and organizational differences should be carefully considered to ensure appropriate adaptation and implementation [83]. For example, because Dutch healthcare professionals attend the custody facilities generally on an on-call basis, the framework explicitly incorporates the role and training of police in recognizing healthcare needs and monitoring detainees. The framework also distinguishes between the financing structures of healthcare provision and medication dispensing, since in the Dutch system medication provided in custody is sought to be billed through the detainees’ health insurance. The presence or absence of structures to fund medication for detainees without health insurance may influence access to care and the quality of care provided. Financial and organizational structural features may also affect workforce organization, triage practices, professional autonomy, and the relationship between police and healthcare professionals. Nevertheless, the framework was designed to capture the broad themes relevant to healthcare in custody, and its overarching domains are expected to be applicable across police custody systems in other countries.

### Strengths and limitations

This study is limited by several aspects. The search strategy for the literature was complicated by differences in terminology and variations in custody settings between countries. Literature about quality of care could have been missed by including only literature published in English or Dutch language. Besides, although the screening process was conducted with great care and any doubts were reviewed twice, some relevant information may still have been missed in the literature because not all literature was independently screened by two authors. However, the inclusion of medical guidance documents and searches of grey literature helped mitigate the potential loss of information. Moreover, we consulted experts to enrich the synthesis of the evidence, as also endorsed by Arksey & O’Malley [16]. The broad set of involved experts indicated that all major factors were included in the framework. Furthermore, involving a diverse set of experts from various backgrounds in the expert consultations, enriched the development process of the conceptual framework by discussing the interplay of different system components in this multi-stakeholder care setting.

## Conclusions

This study provides a conceptual framework that offers a structured approach for assessing and improving healthcare quality in police custody settings. By addressing the unique challenges of short-term detention and integrating insights from multiple sources, the framework offers a valuable tool for enhancing care quality and ensuring that detainees receive equitable and effective healthcare. The development and use of quality indicators based on this framework have the potential to generate valuable knowledge and drive meaningful improvements in healthcare delivery within police custody settings, ultimately benefiting detainees and contributing to broader public health goals.

## Supplementary Information

Supplementary Material 1.

Supplementary Material 2.

Supplementary Material 3.

Supplementary Material 4.

Supplementary Material 5.

## Data Availability

No datasets were generated or analysed during the current study.
