# Supplementary material for: Defining quality of healthcare in Dutch police custody: the development of a conceptual framework for monitoring care quality through a scoping review and expert consultations
Source: BMC Public Health. 2026 Jul 2;26:2128. doi: 10.1186/s12889-026-27949-2 (PMC13359842; doi:10.1186/s12889-026-27949-2)
Supplement: Supplementary file 1 — Supplementary Material 1. [file 12889_2026_27949_MOESM1_ESM.pdf]

Example of search strategy in Medline (Ovid)

1. (health care or health needs or health service* or health status or health outcome* or medical care or medical examination or forensic nurs* or forensic physician or forensic medicine or primary care).tw
2. (quality indicator* or quality measure* or performance measure* or quality metric* or performance metric* or performance indicator* or (quality adj2 (care or healthcare))).tw
3. Quality Indicators, Health Care/
4. Quality of Health Care/
5. 2 or 3 or 4
6. (custody or detention or detain* or arrestee or arrested or watch house or jail).tw
7. (1 or 5) and 6
8. Limit 7 to (yr="2008-Current" and (dutch or english))
