# Supplementary material for: Defining quality of healthcare in Dutch police custody: the development of a conceptual framework for monitoring care quality through a scoping review and expert consultations
Source: BMC Public Health. 2026 Jul 2;26:2128. doi: 10.1186/s12889-026-27949-2 (PMC13359842; doi:10.1186/s12889-026-27949-2)
Supplement: Supplementary file 2 — Supplementary Material 2. [file 12889_2026_27949_MOESM2_ESM.pdf]

Supplementary Table S1: Summary of peer-reviewed scientific publications included in the review (n = 69)

Author(s), year	Aim	Design and data source	Setting, country	Study population	Main conclusion
Arends, de Haan & van 't Hoff, 2009 [66]	To describe the content of the first Dutch national guideline on pharmacological care for detained addicts	Guideline development based on a questionnaire, literature search of guidelines and a national conference	Penal institutions, the Netherlands	Detained opioid addicts	Continuity of care and after-care immediately after detention and optimalization of medical information transfer are crucial (e.g. continuation of methadone maintenance treatment for opioid addicts).
Baksheev, Thomas & Ogloff, 2012 [48]	To examine the predictive power of personal factors (pre-prison characteristics, e.g. psychiatric diagnosis, history of psychiatric treatment), situational factors (environmental effects of police cell conditions), and the interaction of both to account for the occurrence of psychopathology among police cell detainees	Cross-sectional observational studies based on questionnaire assessment tools, and data from a psychiatric case register (public mental health database)	Police custody, Australia	Detainees in police custody aged 18 or older who were willing to participate (n=150)	Those with preexisting vulnerabilities suffer the most in police cells due to the detrimental conditions of the police cells.
Beaufrère & Chariot, 2015 [25]	To determine the proportion of detainees over age 60, their medical characteristics, addictive behaviors, medical history and perceived health status	Retrospective descriptive study of medical examination data	Police custody, France	All arrestees over age 60 examined by forensic physicians (n=211; 2% of total detainee population, accounting for 347 medical examinations), compared to those under age 60 (n=13.041 detainees, accounting for 18.751 medical examinations)	10% of the examined individuals over 60 were declared unfit to be detained, which is a higher proportion than in those under 60 (1%).
Beaufrère, Belmenouar & Chariot, 2014 [24]	To determine the proportion of detainees over age 60, their health characteristics and conditions of detention, and the factors included in medical decisions on fitness to be detained	Retrospective descriptive study of medical examination data	Police custody, France	All arrestees over age 60 examined by forensic physicians (n=180; 1% of total detainee population, accounting for 265 medical examinations)	Elderly individuals commonly present with chronic health disorders and are currently receiving treatments. Unfamiliarity with detained elderly individuals by custody officers can increase the risks associated with detention.

Author(s), year	Aim	Design and data source	Setting, country	Study population	Main conclusion
Bendelow, Warrington, Jones et al., 2019 [74]	To investigate the complexities underlying high rates of police detention under the Mental Health Act	Retrospective descriptive study of detention data and interviews	Police custody under the Mental Health Act, United Kingdom	Individuals detained under the Mental Health Act (s136) during 2012 (n=1142; interviewed: n=37)	Predominantly, police used the Mental Health Act detention as suicide prevention, of which most took place out-of-hours, when no other services or help were available.
Bennett & Holloway, 2008 [52]	To identify and compare health problems and treatment needs among younger and older arrestees, and to draw out the implications of the findings for health education and prevention	Cross-sectional descriptive study based on survey interviews and urine specimen collection analyses for seven drug types	Police custody, United Kingdom	Arrestees who agreed to be interviewed during custody (n=3135, of whom n=1547 were aged 17-24, and n=1588 were aged > 25). Of them, 90% also provided urine specimens (n=2.833)	Young arrestees experience a wide range of physical and mental health problems. 55% of drug dependent 17-24 year old detainees is currently not in treatment but would like treatment.
Bisseling & Braam, 2009 [78]	To investigate whether the time-limits on collaboration between the emergency mental health services and the police, set in national and regional agreements, had been reached	Retrospective descriptive study of police custody registry data and health information system data of the emergency medical services	Police custody, the Netherlands	Emergency cases attended by both the police and the crisis-team of the emergency mental health services (n=69)	85% of contacts was settled within 6 hours (national criterium), and 20% within 2 hours (regional norm).
Brooker, Tocque, Mitchell et al., 2018 [51]	To report on a health needs assessment of detainees in police custody	Retrospective descriptive registry study on custody and health records; and cross-sectional descriptive based on a questionnaire among detainees, interviews with stakeholders and documentary information from previous inspection reports, and minutes of meetings between the health care provider and the police	Police custody, United Kingdom	Detainees in police custody (registry data, n=16.375), custody detainees seen by healthcare staff (registry data, n=3502), detainees answering to a healthcare satisfaction questionnaire (n=106), interviewed key stakeholders (e.g. custody sergeants, healthcare staff from alcohol, drug and mental health services, and other police staff of all grades and roles, n=26)	Age increased the likelihood of seeing the emergency care practitioner or forensic medical examiner. 21% of respondents stated that had any health concern whist in custody.
Buster, Dorn, Ceelen et al., 2014 [21]	To describe the registered mental health, addiction and social problems and compares them to the self-reported problems among a sample of detainees	Cross-sectional descriptive registry study and interviews	Police custody, the Netherlands	Register data of 17.321 detention episodes, and a sample of 264 interviewed detainees	Detainees show high levels of drug abuse, homelessness and psychopathology and have limited contact with health services.

Author(s), year	Aim	Design and data source	Setting, country	Study population	Main conclusion
Carter & Mayhew, 2010 [45]	To determine a baseline for timing, numbers and case mix of detainees referred to hospital for medical assessment in order to review the effectiveness of existing custody procedures for the management of medical emergencies.	Retrospective descriptive study of a custody record system	Police custody, United Kingdom	Detainees referred to hospital from the police custody centers (n=188)	The health care team were recorded as involved in 80% of the detainees transferred to the hospital.
Ceelen, Dorn, Buster et al, 2012 [22]	To examine the medical diagnoses and medicinal treatment of detainees seen in police custody by forensic physicians and nurses and to compare them to the general population	Retrospective descriptive registry study based on custody medical records and records of general practitioners, and a health interview survey among detainees	Police custody, the Netherlan ds	Randomly selected detainees in police custody who participated in a survey (n=264), detainees assessed by the forensic medical service with somatic reasons for consultation (n=4396), and for related prescriptions (n=4912)	Compared to the general population, diabetes mellitus, asthma, HIV/AIDS, epilepsy, lacerations, pain and musculoskeletal problems were among male detainees more often the reason for consultation by the forensic medical service.<br>Among female detainees, hypertension, diabetes mellitus, asthma, HIV/AIDS, musculoskeletal complaints and pregnancy-related problems were frequently the reason for consultation as compared to the general practitioner population.
Chariot, Beaufrère, Denis et al., 2014 [27]	To present the medical characteristics of detainees kept in police custody, including addictive behaviors, traumatic injuries and high-risk medical situations in the context of custody	Prospective descriptive registry study based on a standardized questionnaire for medical evaluation	Police custody, France	All patients aged 13 or older in police custody who were examined by a physician for the assessment of fitness for detention (n=19.098 medical examinations on 13.317 individuals)	Attending physicians should pay particular attention to addictive behaviors and to recent traumatic lesions, both for immediate care and for the prevention of alcohol and psychoactive substance-related harm.

Author(s), year	Aim	Design and data source	Setting, country	Study population	Main conclusion
Chariot, Briffa, Lepresle et al., 2013 [77]	To develop a document that could guide the attending physician to carry out medical examinations, including assessment of fitness for detention	Template development	Police custody, France	Aimed for physicians (forensic physicians, general practitioners, emergency doctors) who attend detainees in custody suites or in hospitals	The developed medical certificate template protects the interests of the examined person in cases of poor conditions of arrest or detention, protects doctors in cases of legal proceedings, and allows epidemiological data to be collected.
Chariot, Lepresle, Lefèvre et al., 2014 [26]	To characterize addictive behaviors in detainees and to evaluate the feasibility of a brief intervention at the time of the medical examination in police custody	Descriptive study of standardized questionnaire for medical evaluation, and prospective feasibility study of brief intervention	Police custody, France	Arrestees aged 15 or older (n=1000) who were examined by a physician for the assessment of fitness for detention	Addictive disorders affected the majority of individuals in police custody.
Chariot, Martel, Penneau et al., 2008 [61]	To present the main points of the statement on guidelines for doctors attending detainees in police custody made during a consensus conference.	Consensus conference based on panel views	Police custody, France	Detainees in police custody	Doctors should prescribe any ongoing treatment that needs to be continued and any emergency treatment that is required. Custody officers may monitor the detainee and administer medication, but should not be expected to exceed that required of the detainee’s family under normal circumstances. Doctor’s opinion should be given in a national standard document.
Coudert, Vidal, Lefèvre et al., 2019 [23]	To describe the characteristics of adolescents under 18 detained in custody	Retrospective descriptive study of standardized questionnaire for medical examination	Police custody, France	Arrestees in police custody examined by physicians aged 13-17 (n=1859)	The detention of adolescents in police stations is commonly associated with assaults at the time of arrest. High proportions of adolescent arrestees smoke tobacco or cannabis.
Cummins, 2008 [59]	To analyze police responses to incidents of self-harm by individuals while in custody	Retrospective descriptive study of police records about incidents of self-harm	Police custody, United Kingdom	Incidents (n=168) of self-harm that occurred in the custody	Alcohol or substance misuse was identified as a clear risk factor for incidents of self-harm in police custody.

Author(s), year	Aim	Design and data source	Setting, country	Study population	Main conclusion
Dembo, Faber, Cristiano et al., 2017 [73]	To determine if there are gender differences in health risk behavior among youth, and if so, if they differ in regard to their sociodemographic and psychological factors and the intervention / service delivery implications of the differences	Retrospective descriptive study of medical intake data	Juvenile assessment center (intake facility) health coach services, United States	Youth aged between 12-17 participating in health tests at central intake facility health coach service (n=779, 77% participation rate)	Heterogeneity and co-occurrence in health risk behaviors (i.e. high levels of frequency of sexual partners, STD positive rates, positive urinalysis rated for marijuana and moderate to severe drug problems) is important to consider.
Dorn, Ceelen, Buster et al., 2014 [43]	To describe mental health problems for which primary care services were provided during police custody, and to shed light on differences in reasons for consultations of police detainees as compared to patients in general practice	Retrospective descriptive study of electronic health records of police custody and general practice data	Police custody, the Netherlands	Detainees seen by the forensic medical service for psychological problems (n=3232), compared to general practice patients aged 20-60 years old (n=91.215)	Among those obtaining medical attention in the police cell, almost half were seen due to mental health problems, with substance abuse as the leading reason for consultation. Twice as many patients from the detainee population received nervous system medication.
Dorn, Janssen, de Keijzer et al., 2018 [36]	To describe the percentage of the consultations of the forensic medical services that result in hospital referrals and the characteristics of the referrals	Retrospective descriptive study of electronic registrations in the forensic medical department.	Police custody, the Netherlands	Detainees who have been referred to emergency departments for further evaluation or emergency care while in police custody (n=244)	Leading reasons for indications associated with referral were injuries (66%), intoxication / withdrawal (11%) and cardiac problems (7%). Hospital admission was the consequence in 18% of the referrals. A minority of referrals was considered unnecessary (7%).
Fablet & Chariot, 2018 [32]	To evaluate the practical implementation of police detention of children aged 10-12 years and to describe their medical characteristics and history, perceived health status and opinion on custody	Retrospective descriptive study of data from a standardized questionnaire during medical examination	Police custody, France	All arrestees younger than 13 years of age (10-12 years) who were detained in police custody (n=76 detention episodes, 0.1% of total examined arrestees)	Progress is needed to improve the care of children suspected of serious crimes, which could include a dedicated space made available for the short-term detention of children in the main police stations.

Author(s), year	Aim	Design and data source	Setting, country	Study population	Main conclusion
Gahide, Lepresle, Boraud et al., 2012 [42]	To describe the frequency of recent traumatic injuries observed at the time of medical examination and to record detainees' self-reported of perceived physical violence before, at the time of, or during custody	Prospective descriptive study of data from a standardized questionnaire during medical examination	Police custody, France	All detainees for whom information regarding allegations of assaults or the presence of traumatic injuries was available (n=2694 detainees)	The attending physician should systematically note detainees' self reports of assaults and investigate recent traumatic injuries, as injured detainees were more frequently declared unfit for detention than non-injured non-assaulted detainees.
Gandon, Outh-Gauer & Chariot, 2018 [38]	To describe the health status of the female population in police custody, identify their specific medical needs and compare the health status of female arrestees to that of males in custody	Retrospective descriptive study of data from a standardized questionnaire during medical examination	Police custody, France	Women aged 13 or older in police custody (n=438; 5% of examined detainee population during study period) who were examined by a physician for the assessment of fitness for detention, the description of traumatic injuries or illicit drug testing, compared to male detainees (n=6970).	Detention in police custody involves a minority of females, who are older and more frequently report somatic or psychiatric disorders.
Gérardin, Guigand, Wainstein et al., 2017 [41]	To provide a description of social and medical characteristics of people in custody, to identify the uses of psychoactive substances and to characterize the problematic use of the psychoactive substances	Observational descriptive study of a questionnaire about detainees' state of health in addition to the medical examination	Police custody, France	People detained in police custody who were required to undergo a medical examination and who admitted to taking at least one illicit drug use and/or one psychoactive medication use (n=882)	Psychoactive substances mentioned by respondents were not different from those observed in the general population, but for certain users, the desired effects were far from the pharmacologically expected ones. Problematic users presented severity criteria which seemed to be greater than in psychoactive substance users in the general population.
Heide, Chariot, Green et al., 2018 [83]	To define the current status of the medical aspects of police custody in the European Union and to set a baseline for establishing future improvements	Cross-sectional descriptive study based on survey data	Police custody in 25 European countries	Detainees receiving police custody care in 25 European countries	Detainees are at higher risk of complications of poor management of healthcare issues due to an over-representation of drug, alcohol, mental health, poorly controlled physical health issues, combined with possibly being subject to different forms of restraint and control.

Author(s), year	Aim	Design and data source	Setting, country	Study population	Main conclusion
Heide, Kleiber, Hanke et al., 2009 [57]	To describe deaths in custody cases	Retrospective study of post-mortem records	Death in police custody, Germany	Persons died in police custody (n=60)	In 27% of cases, death was very probably not preventable even if the detainee had received all necessary care. In the majority of the studied cases, partly blatant deficiencies were found in the conduct of doctors and policemen and there would have been good chances to prevent a fatal outcome if the persons would have been admitted to hospital early enough.
Heide, Stiller, Lessig et al., 2012 [62]	To view the current situation of (legal regulations of) medical assessments, analyze whether these have a significant effect on the medical aspects of police custody, and provide practical recommendations for police officers and doctors, and propose changes in the legal conditions	Retrospective descriptive study of medical records	Police custody, Germany	Detainees who underwent an fitness for police custody examination (n=3.674)	The recent introduction of new police custody regulations in the studied region had a significant influence on the medical decision on fitness for custody.
Kennedy, Payne-James, Payne-James et al., 2022 [110]	To explore the frequency and nature of complaints against healthcare providers in the police custodial and sexual assault referral centers settings, and the mechanisms by which they are investigated	Complaints against healthcare professionals. Information requested from: police services, regulatory bodies of healthcare professionals, and the Independent Police Complaints Commission	Police custody, United Kingdom	39 police services (out of 44 requested to participate), complaints of 32 individual cases	The majority of police services did not or could not provide information about complaints against healthcare professionals. The most frequently cited explanation was that healthcare provision was contracted to private companies and therefore complaints were primarily a matter for the companies through which they were employed.
Kuchewar, Bhosle, Shrigiriwar et al., 2020 [56]	To analyze the different causes of custody-related deaths and associated factors and to identify the areas of intervention for prevention of such deaths	Retrospective descriptive study of files about custody-related deaths	Death in custody, including police custody, India	Autopsies after custody-related deaths (police custody: n=12)	Avoidable reasons for custody-related deaths: lack of knowledge regarding the causes of death, careless attitude towards the suicidal behavior, and the health and welfare of detainees.

Author(s), year	Aim	Design and data source	Setting, country	Study population	Main conclusion
Leese & Russell, 2017 [60]	To explore how custody staff in different roles within the organization worked to safeguard vulnerable people in custody	Cross-sectional descriptive study based on semi-structured interviews	Police custody, United Kingdom	Custody staff (n=10)	The respondents expressed frustration that vulnerable people find themselves in police custody for low-level crime, when it could have been avoided with improved mental health services in the community. Despite the processes that are designed to safeguard the detainee, tensions still exist including, timely access to mental health assessments, appropriate training and support for staff, and the use of appropriate adults.
Lepresle, Mahindhoratep, Chiadmi et al., 2012 [105]	To determine medical characteristics and addictive behaviors of detainees held in police custody for drink-driving and to collect data regarding reported assaults or observed injuries in these individuals	Prospective descriptives study based on medical examination data, including outcome of a breath alcohol test	Police custody, France	All patients aged 18 or more held in police custody for proven (n=223) or suspected (n=55) driving under the influence of alcohol and examined by a physician for assessment of fitness for detention, compared to controls who were detained for other reasons than suspicion of drink-driving under the influence of alcohol (n=2212)	Physicians need to give attentive care to detained drink-drivers. Special attention should be paid to drink-drivers who refused or were not able to complete breath alcohol measurement.
Ma, Du, Cai et al., 2016 [72]	To identify challenges of community-based drug dependence treatment associated with the role of the police and examine how the role of police in collaboration, approach and performance indicators could contribute to these challenges and how changes in these might improve community-based drug dependence	Cross-sectional descriptive study based on semi-structured interviews	collaboration between community-based drug dependence rehabilitation centers and police service bureau, China	Interviews with staff of an community-based drug dependence treatment center (n=4), police officers N=3), center for disease control (n=3), and people who use drugs (n=16)	To overcome barriers to effective community-based drug treatment, we recommend aligning the goals of law enforcement and public health agencies towards health-based performance indicators.

Author(s), year	Aim	Design and data source	Setting, country	Study population	Main conclusion
Magee, Fortenberry, Rosenman et al., 2021 [49]	To examine the prevalence of mental health and co-occurring substance use disorder diagnoses among arrestees, examine whether this is higher among those with repeat arrests, and to identify opportunities for intervention for nonviolent offenses	Retrospective descriptive cohort study based on arrest data from the police and clinical data from a regional health exchange that contains patient-level medical records	Arrestees , United States	All individuals arrested over the course of one year (n= 22.939 individuals; n=30.301 arrests)	The results suggest individuals with co-occurring mental health and SUD diagnoses have a greater likelihood of repeat arrest.
Mahamad Arif, Syed Alwee, Shafee et al., 2021 [54]	To provide a general overview of the pattern including the causes and the demographic profile involving death in custody	Retrospective descriptive registry study	Four custodial settings including police custody, Malaysia	Persons who died under the custody of any enforcement agency, including police custody (n=30)	Detainees under police custody showed the least number of deaths from natural causes, compared to the prison, immigration depot for illegal immigrants and the army lock-up.
Mahindhoratep, Lepresle, Chiadmi et al., 2013 [79]	To evaluate medical features and addictive behaviors of suspected drug drivers and to collect data regarding reported assaults or observed injuries in these individuals	Prospective descriptive study based on medical examination data	Hospital forensic medicine unit, France	Patients aged 18 or over held in police custody examined by a physician for assessment of fitness for detention or for urine testing or blood sampling, and for whom a laboratory of forensic toxicology was requested for blood testing. Divided in groups of: drug drivers (tested positive for drugs in urine and blood, n=205), wrongly suspected drug drivers (tested positive in urine and negative in blood, n=116), drink drivers (evaluated by alcohol blood testing, n=231), and a control group (held in custody for other reasons, n=2427)	Arrested drug drivers were young, healthy, and infrequently reported assaults or presented traumatic injuries, which does not put them in a high risk medical condition.

Author(s), year	Aim	Design and data source	Setting, country	Study population	Main conclusion
McKenna, Murphy, Rosenbrier et al., 2019 [69]	To describe the characteristics of police custody detainees who were referred to the Trust Criminal Justice Liaison and Diversion Service, with a focus on their mental health needs, in order to compare and contrast with other Liaison and Diversion services	Retrospective descriptive study based on a Liaison and Diversion dataset	Police custody and Liaison and Diversion Service, United Kingdom	Detainees with complex health needs, substance misuse and vulnerabilities requiring specific intervention who were referred to a criminal justice liaison and diversion service of a police station (n=858)	The Liaison & Diversion service saw a small number of referrals compared to arrests during the period (4.7% referral rate), but the acceptance rate of assessment by detainees referred was high (86%).
McKinnon & Grubin, 2010 [40]	To determine the extent of health problems and 'mental vulnerability' in detainees in police custody, and to determine the efficacy of current health screening procedures	Cross-sectional descriptive study based on police arrest records	Police custody, United Kingdom	Detainees who were referred to the forensic medical examiner (n=307)	The current police screening procedure detects only a proportion of the amount of health morbidity of the detainees in police custody.
McKinnon & Grubin, 2013 [47]	To evaluate the efficacy of police screening procedures	Prospective descriptive study based on clinical interviews with custody detainees	Police custody, United Kingdom	Detainees in police custody who agreed to participate in the study (n=237)	Given the amounts of morbidity and the need for reliable triage, improvement in the health screening procedures used by the police is needed.
McKinnon, Hayes & Grubin, 2017 [44]	To compare characteristics and health care needs of older police custody detainees (>50) with their younger counterparts	Cross-sectional descriptive study based on a questionnaire and (medical) interviews with detainees	Police custody, United Kingdom	Police detainees over 50 (n=57) compared to younger detainees (>18 years) (n=543)	Compared to their younger counterparts, older detainees (>50) had higher rates of physical illness (asthma, diabetes, hypertension, current gastro-intestinal symptoms), were more judged to be at risk of alcohol withdrawal, and were more likely to be taking medication. A higher proportion had presentations consistent with cognitive impairment due to possible dementing processes. Over 80% of them were recommended to have a health assessment in police stations based on their presentation. There were equivalent rates of mental disorder and drug taking compared with younger detainees.

Author(s), year	Aim	Design and data source	Setting, country	Study population	Main conclusion
McKinnon, Thomas, Noga et al., 2016 [103]	To explore the available evidence regarding healthcare issues in police custody by describing the types and prevalence of health disorders encountered in custody and by providing an overview of current practice and recent innovations	Scoping review of literature retrieved from scientific databases	Police health screening in United Kingdom, continent al Europe, North America, Australia	Detainees entering police custody	Research to improve the health of police custody detainees requires greater priority, focusing on case identification and service redesign to address high levels of morbidity and to facilitate health promotion and prevention activities.
Miles, Webb, Kevern et al., 2020 [100]	1) To test the predictive accuracy of a custody early warning score system that helps non-medical detention staff to identify detainee morbidity and mortality risk upon arrival and identifying detainee health need and prioritization. 2) To study how well the tool correlated to another monitoring tool and how accurate those two systems are at pre-empting the medical emergencies and hospital referrals that occur in police custody	Trial of the warning score system using a self-reported health questionnaire	Police custody, United Kingdom	Detainees arriving into police custody (n=1.163)	The assessed scoring systems were not sensitive enough to identify health need in the detainee population, due to frequent, altered physiological parameters. Such scoring systems add little to the risk assessment process, with low scores allowing for complacency and a false reassurance, when using a system designed for very different circumstances.
Morgan, Morgan, Valuri et al., 2013 [50]	To provide reliable data on the association between mental illness and offending	Whole-of-population longitudinal cohort study with record-linked data	Police arrests, Australia	Cohort of people born between 1955-1969 and arrested between 1985-1996 (n=116.656)	The period prevalence of arrest for people with any psychiatric illness was 32.1 %.
Nwafor, Nwarfor, Eziagu et al., 2021 [53]	To identify the peculiarities of death among detained individuals.	Retrospective descriptive study based on autopsy registers, reports sent to the coroner, and post mortem radiological imaging	Police and prison custody, Nigeria	Detainees who died in police or prison custody over a 4-year period whose relatives requested a post mortem examination (n=6 in police custody)	Natural deaths is the most common manner of death among the studied cases.

Author(s), year	Aim	Design and data source	Setting, country	Study population	Main conclusion
Ogloff, Warren, Tye et al., 2011 [35]	To examine the frequency and nature of psychiatric symptoms experienced by persons being held in custody in police cells, to determine the contribution of preexisting psychopathology to current symptoms by examining each detainee's history of contacts with mental health services, and to examine the offences that led to the arrest of the detainees to determine whether differences existed in offending patterns between participants with and without psychiatric problems	Cross-sectional descriptive study based on clinical interviews executed by nurses, data from a public mental health service database, a police database and a general practitioner database	Police custody, Australia	Detainees (n=614)	Many detainees experience past and present psychiatric symptoms, which creates a significant clinical need necessitating timely access to healthcare and a continuity of care with health service providers beyond the initial police cell contact.
Payne-James, Green, Green et al., 2010 [34]	To determine the level of general health issues, diseases and/or pathology for detainees in police custody, and to determine how well those general health issues, diseases and/or pathology are being managed	Descriptive cross-sectional study based on a questionnaire survey for detainees completed by forensic medical examiners.	Police custody, United Kingdom	Detainees in police custody who were seen by forensic medical examiners (n=201)	In part because of the chaotic lifestyle of many detainees, appropriate care was not being rendered, thereby, putting both detainee and potentially others coming into contact with them, at risk.
Rees, 2020 [80]	To explore the ways nurses are able to achieve interagency collaboration with a particular police officer, the Desk Sergeant	Cross-sectional descriptive study based on semi-structured interviews with custody nurses	Police custody, United Kingdom	Custody nurses (n=20)	Nurses accomplish interagency interoperability by interacting regularly with the Desk Sergeant, anticipating their needs and limiting their own goals to those that are commensurate with the Desk Sergeant's, notably providing information and avoiding deaths in custody.

Author(s), year	Aim	Design and data source	Setting, country	Study population	Main conclusion
Rekrut-Lapa & Lapa, 2014 [68]	To review and analyze the literature concerned with the health needs of detainees in police custody	Literature review	Police custody, United Kingdom	Detainees in police custody in England and Wales (n=12.000 total from all included literature)	Mental health problems including substance misuse and physical conditions are highly prevalent among the custody population and require both emergency and routine care. The current quality of the healthcare services has been discussed and the need for improvement has been indicated by a number of agencies.
Samele, McKinnon, Brown et al., 2021 [46]	To examine psychiatric and developmental morbidity and the level of unmet health and social care needs, and ascertain differences in need between morbidity categories amongst police detainees	Cross-sectional descriptive study based on interviews and standardized measures, supplemented by information from police custody records	Police custody, United Kingdom	A sample of detainees in police custody aged 18 or over (n=134)	Amongst police detainees, there are high rates of mental health problems, risk of suicide, and high levels of unmet need, especially as regards accommodation.
Scott, McGilloway, Donnelly, 2009 [84]	To describe the population of female detainees referred to a mentally disordered offenders service	Retrospective descriptive study based on administrative outcomes and psychiatric assessment data	Police custody, United Kingdom	Female detainees in police custody	Many women with mental illness are arrested and detained in police custody. The assessed criminal justice liaison and diversion service effectively identified, assessed and linked the majority of female mentally disordered offenders to services.

Author(s), year	Aim	Design and data source	Setting, country	Study population	Main conclusion
Senior, Noga, Shaw, 2014 [71]	To discuss challenges and opportunities inherent in addressing people's healthcare needs whist in police custody	Descriptive study of scientific literature	Police custody, United Kingdom	Detainees in police custody	The introduction of universal mental health screening for all detainees in police custody is a good place to start, but needs to be accompanied by willingness on the part of government, commissioners, clinicians at all levels of service and the police to tackle honestly, and with credibility and compassion, the issue of what should happen to people with mental disorder who find themselves in contact with the police.
Sirdifield & Brooker, 2012 [31]	To make a health needs assessment which includes an investigation into the health needs of detainees and an assessment of any gaps in, or possible improvements to the current model of service provision	Retrospective descriptive registry study based on custody sergeants' risk assessment records, medical examination records, and interviews with police and health care staff	Police custody, United Kingdom	A random sample of detention records (n=1917), and interviews with key police and healthcare staff (n=52)	Detainees experience a wide range of health problems requiring active management during detention.
Sondhi, Williams, 2018 [28]	To examine the extent and nature of health needs in police custody	Descriptive cross-sectional study based on a survey answered by healthcare providers about the examined detainees	Police custody, United Kingdom	Detainees in police custody who are assessed by healthcare providers (n=1657)	There is a need for police custody staff to consider detainees' dual diagnosis needs. The development of integrated interventions alongside the enhanced clinical management of alcohol, drug use and mental health was considered.
Struijk, 2012 [64]	To discuss current developments in temporary holding cells in police custody, in terms of their implications for housing, security, care and treatment of detainees in police custody	Descriptive study based on literature	Police custody, the Netherlands	Police custody cell facilities and developments	Developments in the field of police custody highlight the importance of an (active and constructive) thinking supervisory (audit) committee.

Author(s), year	Aim	Design and data source	Setting, country	Study population	Main conclusion
Sturgiss, Parekh, 2011 [37]	To determine characteristics of detainees seen by forensic physicians and the type of work performed by the forensic physicians	Retrospective clinical audit based on the database of forensic physicians	Watchhouse, Australia	Detainees seen by forensic physicians (n=988 occasions), and the type of work performed by forensic physicians in a watchhouse	The majority of the workload in the studied watchhouse involves medical review of detainees and not forensic procedures. As many detainees gave histories of mental health and/or drug and alcohol problems, it is essential that forensic physicians are skilled in these areas.
Sufrin, Tulsky, Goldenson, 2010 [107]	To describe the proportion and characteristics of newly arrested women who are eligible for and interested in taking emergency contraception	Cross-sectional descriptive study of survey results	(Women) jail intake facility, United States	Newly arrested women between the ages 18 and 44	48% of the eligible women indicated a willingness to take emergency contraception if offered.<br>71% percent of all women indicated that they would accept an advance supply of emergency contraception upon release from jail.
Thomas & Forrester-Jones, 2019 [75]	To re-examine the types of behaviors leading to detention and to examine the circumstances and factors which caused officers to use their power of detention	Cross-sectional descriptive study of police records of mental illness, health trust data (covering health assessments after a detention under the Mental Health Act), and semi-structured interviews with police officers	Police detention under the Mental Health Act, United Kingdom	Incidents officers were sent to in where mental illness or disorder was a principle concern (n=660). Interviewed police officers: n=17	Around 0.6% of the calls from the public to the police force related to mental illness.
Thoonen, Kubat & Duijst, 2015 [58]	To describe case files regarding police custody deaths in the Netherlands	Retrospective descriptive study of medical investigation results, medico-legal post mortems (if performed) from the police files and from the Dutch Forensic Institute, and Public Prosecutor files	Death in police custody, the Netherlands	All cases of death in police custody (78 case files) relating to police custody deaths in the Netherlands	Half of the calamities occurred during the period of apprehension and transport by the police. The consumption of drugs and/or alcohol seems to be an important factor in fatal medical emergencies occurring under the responsibility of the police.

Author(s), year	Aim	Design and data source	Setting, country	Study population	Main conclusion
Van den Hondel, Saaltink & Bender, 2016 [30]	To provide an overview of the outcomes of psychiatric assessments of apparent mentally ill detainees in police stations	Retrospective descriptive study of medical examination data	Police custody, the Netherlands	Detainees who received a psychiatric assessment in police cells (n=9669 psychiatric assessments, 19% of total episodes)	Many apparent mentally ill individuals brought to the police station are sent home by the forensic physician. Before the psychiatric assessment, medical causes of psychiatric illnesses, for example excited delirium syndrome and hypoglycemia, drug use (GHB, cocaine, heroin), and cerebral pathology are excluded.
Verdier, Denis, Bourokba, 2018 [39]	To describe the health and social characteristics of detainees in police custody as compared to the general population of the same region	Cross-sectional descriptive study based on a prospective sample of all examined arrestees, based on survey data asked by forensic physicians during medical examination	Police custody, France	Detainees in police custody (n=600) aged over 18 who were seen by forensic physicians and agreeing and able to participate, compared to a random sample of adults of the general population (n=3000)	Comparative analysis of male arrestees and males from the general population showed that the male arrestees had worse social and health conditions.
Vidal, Lepresle & Chariot, 2017 [29]	To describe the characteristics of arrestees reporting mental health issues and the assessment of fitness to be detained in these individuals	Cross-sectional descriptive study of self-reported health issues by a detainee to a physician via additional questions to a standardized questionnaire during medical examination	Police custody, France	Detainees (n=420) who reported having a current mental health issue, compared to detainees with no mental health issue (n=4394)	The high proportion of patients with interrupted mental healthcare among those reporting mental disorders suggests that the medical examination during custody could be a significant opportunity to restore psychiatric care.
Vincent, Beaufrère & Chariot, 2015 [33]	To describe the characteristics of detainees arrested for the first time and who had medical examination during custody	Retrospective descriptive study of a standardized questionnaire for medical examination	Police custody, France	Detainees aged 13 or over arrested who had a medical examination during custody. Groups were divided in those who: later on were examined a second time (n=200), those who were not examined a second time (randomly selected n=200), and a control group of who were arrested before (randomly selected n=200)	The findings suggested only minor differences between clinical features of individuals arrested for the first time and their clinical status when they were arrested for the second time.

Author(s), year	Aim	Design and data source	Setting, country	Study population	Main conclusion
Visscher, van de Kraats, van der Goot, et al., 2015 [70]	To determine what procedure (prison or clinic) will provide the suspect with appropriate care; to find out how to ensure that mentally-ill perpetrators of offences are not assigned erroneously to psychiatric care and thereby exempted from prosecution, and to determine whether the term 'suited for detention' is applicable to such a person	Cross-sectional descriptive study based on semi-structured interviews	Police custody and acute mental health services, the Netherlands	Professionals involved in the assessment of suspects with psychiatric issues who need to be evaluated by mental health services at the police station (n=19).	The role of the representative of the emergency services present at the police station is to decide what type of care is required at that particular moment. Awareness of the importance of a dual track policy (conviction/punishment or care) should help to prevent unjustified 'psychiatrisation', of both minor and serious offenses.
Vohra, Singh & Malik, 2016 [55]	To examine cases of custodial deaths in detail	Prospective study of cases of custodial deaths brought for postmortem examination	Death in custody, including police custody, India	Cases of custodial deaths brought for postmortem examination to the department of forensic medicine and toxicology (police custody: n=1)	The one death in police custody was due to natural cause.
Wainwright & Mojtahedi, 2020 [81]	To examine differences in casual attributions and stereotypes of individuals experiencing mental illnesses, mainly, schizophrenia between police custody staff and members of the general population	Cross-sectional descriptive study based on a self-administered attribution questionnaire	Police custody, United Kingdom	Police custody staff (n=77) and members of the general population (n=85)	The general population held more negative attitudes towards individuals experiencing mental illnesses than police custody staff. In particular they endorsed the attributions anger, avoidance, dangerousness and fear. Custody staff were found to help vulnerable adults more than the general population.
Walker, Higgs, Stoové et al., 2019 [63]	To attend to the perspectives and experiences of young men with histories of injecting drugs about police custody	Cross-sectional descriptive study based on interviews	Police custody, Australia	Young men, aged 18-24, with histories of injecting drug use who were detained in police custody prior to their most recent incarceration in adult prison (n=28)	The positioning of young men with histories of injecting drug use who were detained in police custody prior to adult prison allowed forfeiting of their rights to basic healthcare, fair treatment and respect, and produced and exacerbated a range of psychological, physical and social harms.

Author(s), year	Aim	Design and data source	Setting, country	Study population	Main conclusion
Wardrop, Ranse, Chaboyer et al., 2021 [19]	To synthesize the critically appraise the evidence on healthcare delivered to detainees in short-term custody settings	Scoping review	Police custody in: United States, Australia, Europe and Canada	Detainees in short-term custody settings	Gaps in evidence identified limited international perspectives as most studies originated from the United States, a focus on detainee conditions/symptoms and limited long-term research within the area of health care in short-term custodial settings. Literature regarding health care delivery in short-term custodial settings focused predominantly on mental illness identification.
Webb, Stark, Cutts et al., 2010 [67]	To discuss the implementation of clinical risk management within the custody setting	Retrospective descriptive study based on the call-handler computer system and notes (e.g. from proforma, police documentation, NSPIS records).	Police custody, United Kingdom	Clinical incidents, near miss or positive intervention (n=159 out of 86,184 patient/detainee episodes)	It is recognized that the figures under represent how many critical incidents may have occurred.
Williams, Norman & Sondhi, 2019 [82]	To present the voices of police practitioners involved in custody suites and to provide an overview of their perceptions about the current methods used to deal with mental healthcare needs	Cross-sectional descriptive study based on interviews with key practitioners who work within the custody environment to support detainees, and observations in custody suites	Police custody, United Kingdom	Police practitioners (police personnel, custody sergeants, and dedicated detention officers) involved in custody suites (n=37)	Various notions of risk are central within police custody. Current practices are not sufficient for ensuring the safety of both detainees and officers.

Author(s), year	Aim	Design and data source	Setting, country	Study population	Main conclusion
Wright, Barclay, Frier et al., 2008 [65]	To evaluate how diabetes was being managed in people being detained by the police, and to evaluate a liaison initiative between a diabetes service and the custodial division	Evaluation of current practices based on observations and meetings	Police custody, United Kingdom	Police offers who have to manage detainees with diabetes	The liaison initiative led to the implementation of training for police staff to deal with people with diabetes who require insulin therapy, in blood glucose monitoring and in the management of diabetic emergencies. Equipment for diabetic management in the police station was upgraded, and training provided for its use. Police awareness of the potential problems associated with diabetes was heightened.
