# Supplementary material for: Defining quality of healthcare in Dutch police custody: the development of a conceptual framework for monitoring care quality through a scoping review and expert consultations
Source: BMC Public Health. 2026 Jul 2;26:2128. doi: 10.1186/s12889-026-27949-2 (PMC13359842; doi:10.1186/s12889-026-27949-2)
Supplement: Supplementary file 3 — Supplementary Material 3. [file 12889_2026_27949_MOESM3_ESM.pdf]

Supplementary Table S2: Healthcare providers' activities and advices regarding examined people detained in short-term police custody settings

Health(care) category	Age (years) and/or gender*	Percentage of study population	Of total detainees	Information source	Publication
**Medical examination requests**					
Total registered contacts with a healthcare professional in police custody as percentage of episodes of detention	All	24%	arrested	Medical examination record	[21]
	All	27%	arrested	Medical examination record	[22]
	All	48%	arrested	Police custody record	[40]
	< 25	13%	arrested	Medical examination record	[21]
	25-34	21%	arrested	Medical examination record	[21]
	35-44	29%	arrested	Medical examination record	[21]
	≥ 45	37%	arrested	Medical examination record	[21]
	Men, all	23%	arrested	Medical examination record	[21]
	Women, all	32%	arrested	Medical examination record	[21]
Referred to healthcare provider by custody officer as percentage of all detainees	18-49	69%	arrested	Police custody record	[44]
	≥ 50	52%	arrested	Police custody record	[44]
Recommendation of researchers that detainee should be reviewed by a healthcare provider as percentage of all detainees	18-49	53%	arrested	Interview with researcher	[44]
	≥ 50	82%	arrested	Interview with researcher	[44]
Medical examination requested by detainee as percentage of all medical examinations	13-15	20%	arrested	Medical examination record	[23]
	All	37%	examined	Medical examination record	[42]
	16-17	35%	examined	Medical examination record	[23]
	< 60	40%	examined	Medical examination record	[25]
	≥ 13	31-51%	examined	Medical examination record	[33]
	≥ 13	40%	examined	Medical examination record	[27]
	≥ 60	36%	examined	Medical examination record	[25]
	Women ≥ 13	39%	examined	Medical examination record	[38]
Detainee did not report any health problems, but physician identified ongoing medical problem	≥ 15	3%	examined	Medical examination record	[26]
**Reason for healthcare professional call-out**					
Alcohol	All	16%	examined	HCP activity questionnaire	[28]
Drunk	All	8%	examined	Medical examination record	[31]
Drink/drug driving	All	1%	examined	HCP activity questionnaire	[28]
Drugs	All	3%	examined	Medical examination record	[31]
	All	15%	examined	HCP activity questionnaire	[28]
Fitness to be detained/interviewed	All	12%	examined	HCP activity questionnaire	[37]
	All	41%	examined	Medical examination record	[40]
	All	69%	examined	Medical examination record	[31]
	All	70%	examined	HCP activity questionnaire	[28]
	≥ 16	98%	study participants	Questionnaire	[34]
Fit to charge and release	≥ 16	11%	study participants	Questionnaire	[34]
Injury	All	1%	examined	HCP activity questionnaire	[37]
	All	13%	examined	Medical examination record	[31]
	All	22%	examined	HCP activity questionnaire	[28]
Medical review	All	82%	examined	HCP activity questionnaire	[37]
Mental health	All	11%	examined	Medical examination record	[31]
	All	20%	examined	HCP activity questionnaire	[28]
	≥ 16	10%	study participants	Questionnaire	[34]
Medication	All	21%	examined	HCP activity questionnaire	[28]
	All	21%	examined	Medical examination record	[31]
Other	All	12%	examined	HCP activity questionnaire	[28]
**Mental health and psychiatric assessments**					

Psychiatric assessment / opinion	All	2%	examined	Questionnaire	[29]
	All	19%	arrested	Medical examination record	[30]
Referred to public mental health care after psychiatric assessment	All	48%	assessed	Medical examination record	[30]
Assessed to be at current risk of self-harm/suicide	All	4%	examined	Medical examination record	[31]
**Fitness for custody**					
Outcome of fitness for custody assessment:					
Unconditionally fit for custody	All	84%	assessed for fitness for custody	Medical examination record	[42]
	All	85%		Medical examination record	[29]
	≥ 13	75-89%		Medical examination record	[33]
	< 60	80%		Medical examination record	[25]
	10-12	93%		Medical examination record	[32]
	13-17	92%		Medical examination record	[23]
	13-15	94%		Medical examination record	[23]
	16-17	91%		Medical examination record	[23]
	≥ 60	30%		Medical examination record	[25]
	Women ≥ 13	92%		Medical examination record	[38]
Unfit for custody	All	1%	assessed for fitness for custody	Medical examination record	[29]
	All	1%		Medical examination record	[29]
	All	2%		Medical examination record	[42]
	≥ 13	0-1%		Medical examination record	[33]
	≥ 13	1%		Medical examination record	[27]
	< 60	1%		Medical examination record	[25]
	10-12	0%		Medical examination record	[32]
	13-17	1%		Medical examination record	[23]
	13-15	<1%		Medical examination record	[23]
	16-17	1%		Medical examination record	[23]
	≥ 60	10%		Medical examination record	[25]
	Women ≥ 13	<1%		Medical examination record	[38]
Fit for custody, subject to a variety of conditions	All	14%	assessed for fitness for custody	Medical examination record	[29]
	All	40%		Medical examination record	[29]
	≥ 13	7-19%		Medical examination record	[33]
	< 60	15%		Medical examination record	[25]
	10-12	4%		Medical examination record	[32]
	13-17	4%		Medical examination record	[23]
	13-15	2%		Medical examination record	[23]
	16-17	6%		Medical examination record	[23]
	≥ 60	59%		Medical examination record	[25]
Fit for custody during daytime, but unfit to spend the night	Women ≥ 13	<1%	assessed for fitness for custody	Medical examination record	[38]
Required medical surveillance to remain in custody	≥ 60	58%	assessed for fitness for custody	Medical examination record	[24]
Fit for custody, provided that second medical examination will be performed within 24h	Women ≥ 13	1%	assessed for fitness for custody	Medical examination record	[38]
Surveillance of awareness recommended	≥ 60	37%	assessed for fitness for custody	Medical examination record	[24]
Close surveillance recommended	≥ 60	8%	assessed for fitness for custody	Medical examination record	[24]
	Women ≥ 13	2%		Medical examination record	[38]
Unable to assess fitness for custody	≥ 13	2%	assessed for fitness for custody	Medical examination record	[27]
	13-17	1%		Medical examination record	[23]
	13-15	1%		Medical examination record	[23]
	16-17	1%		Medical examination record	[23]
**Treatment**					
Treatment administered during	10-12	6%	arrested	Medical examination record	[32]

police custody	13-15	22%	arrested	Medical examination record	[23]
	≥ 13	39-53%	examined	Medical examination record	[33]
	≥ 13	50%	examined	Medical examination record	[27]
	16-17	35%	examined	Medical examination record	[23]
	13-17	30%	examined	Medical examination record	[23]
	< 60	44%	examined	Medical examination record	[25]
	≥ 60	53%	examined	Medical examination record	[25]
Active health issues requiring management during custody	≥ 16	56%	study participants	Questionnaire	[34]
Injuries noted/assessed/treated	All	23%	examined	Medical examination record	[31]
Medical management of substance withdrawal	All	22%	arrested	Nursing data	[35]
Transferred to hospital	All	2%	arrested	Police custody record	[45]
	All	1%	examined	Medical examination record	[22]
	All	2%	examined	Medical examination record	[36]
	All	7%	examined	HCP activity questionnaire	[37]
	< 60	1%	examined	Medical examination record	[25]
	≥ 60	3%	examined	Medical examination record	[25]
	≥ 60	1%	examined	Medical examination record	[24]
**Medication during police custody**					
Medication administered / prescribed	All	26%	examined	Medical examination record	[31]
	All	54%	examined	Medical examination record	[42]
	All	63%	examined	HCP activity questionnaire	[37]
	≥ 13	50%	examined	Medical examination record	[27]
	≥ 15	35%	examined	Medical examination record	[26]
	≥ 60	72%	examined	Medical examination record	[24]
	Women ≥ 13	59%	examined	Medical examination record	[38]
Alimentary tract and metabolism problem medication	Men, all	34%	examined	Medical examination record	[22]
	Women, all	26%	examined	Medical examination record	[22]
Antibiotic	All	4%	examined	HCP activity questionnaire	[37]
Antidepressant	All	5%	examined	HCP activity questionnaire	[37]
Anti epileptic	All	3%	examined	HCP activity questionnaire	[37]
Antipsychotic medication	All	5%	examined	Medical examination record	[31]
	All	6%	examined	HCP activity questionnaire	[37]
Benzodiazepine / tranquilizer	All	34%	examined	HCP activity questionnaire	[37]
	All	60%	examined	Medical examination record	[31]
Cardiovascular system medication	Men, all	26%	examined	Medical examination record	[22]
	Women, all	23%	examined	Medical examination record	[22]
Methadone prescription	All	7%	arrested	Medical examination record	[21]
	< 25	1%	arrested	Medical examination record	[21]
	25-34	5%	arrested	Medical examination record	[21]
	35-44	10%	arrested	Medical examination record	[21]
	≥ 45	13%	arrested	Medical examination record	[21]
	Men, all	6%	arrested	Medical examination record	[21]
	Women, all	10%	arrested	Medical examination record	[21]
	All	<1%	examined	HCP activity questionnaire	[37]
Nervous system medication	All	42%	examined	Medical examination record	[43]
Non-opioid analgesic	All	36%	examined	HCP activity questionnaire	[37]
Opioid analgesic	All	33%	examined	Medical examination record	[31]
Painkiller	All	30%	examined	Medical examination record	[31]
	≥ 13	23-29%	examined	Medical examination record	[33]
Psychotropic medication	≥ 13	6-18%	examined	Medical examination record	[33]
Respiratory system medication	Men, all	14%	examined	Medical examination record	[22]
	Women, all	20%	examined	Medical examination record	[22]
Substitution medications for opiate, narcotic or alcohol abuse	All	23%	arrested	Nursing data	[35]

*(Sub)groups other than age and gender not reported in this table
