# Supplementary material for: Defining quality of healthcare in Dutch police custody: the development of a conceptual framework for monitoring care quality through a scoping review and expert consultations
Source: BMC Public Health. 2026 Jul 2;26:2128. doi: 10.1186/s12889-026-27949-2 (PMC13359842; doi:10.1186/s12889-026-27949-2)
Supplement: Supplementary file 4 — Supplementary Material 4. [file 12889_2026_27949_MOESM4_ESM.pdf]

Supplementary Table S3: Identified health problems of people in short-term police custody settings

Health(care) category	Age (years) and/or gender *	Percentage of study population	Of total detainees	Information source	Publication
**Any health problem**					
Any health problem, illness or injury	All	28%	arrested	Police custody record	[31]
	≥ 15	27%	examined	Medical examination record	[26]
	≥ 18	30%	study participants	Questionnaire	[39]
**Somatic health problems**					
Somatic disorder(s)	10-12	18%	arrested	Medical examination record	[32]
	13-15	6%	arrested	Medical examination record	[23]
	All	53%	examined	Medical examination record	[22]
	≥ 18	30%	study participants	Questionnaire	[39]
	16-17	7%	examined	Medical examination record	[23]
	< 60	17%	examined	Medical examination record	[25]
	≥ 60	77%	examined	Medical examination record	[24]
	≥ 60	53%	examined	Medical examination record	[25]
Abdominal pain	All	3%	examined	Medical examination record	[40]
Abdominal symptoms	All	25%	study participants	Interview with psychiatrist	[47]
Anaemia	10-12	2%	arrested	Medical examination record	[32]
Asthma	10-12	2%	arrested	Medical examination record	[32]
	18-49	13%	arrested	Interview with researcher	[44]
	≥ 50	25%	arrested	Interview with researcher	[44]
	All	2%	examined	Medical examination record	[31]
	All	8%	examined	Medical examination record	[40]
	All	9%	examined	HCP activity questionnaire	[28]
	≥ 13	5%	examined	Medical examination record	[27]
	≥ 16	10%	study participants	Questionnaire	[34]
	≥ 18	10%	study participants	Questionnaire	[39]
	≥ 60	2%	examined	Medical examination record	[24]
	≥ 60	3%	examined	Medical examination record	[25]
	Women ≥ 13	9%	examined	Medical examination record	[38]
	Men ≥ 13	5%	examined	Medical examination record	[38]
	All	16%	study participants	Interview with psychiatrist	[47]
Blood-borne virus	18-49	2%	arrested	Interview with researcher	[44]
	≥ 50	4%	arrested	Interview with researcher	[44]
Breathing difficulties	≥ 60	2%	examined	Medical examination record	[24]
Cancer	≥ 60	5%	examined	Medical examination record	[24]
Cardiac	≥ 16	1%	study participants	Questionnaire	[34]
Cardiovascular symptoms	18-49	13%	arrested	Interview with researcher	[44]
	≥ 50	18%	arrested	Interview with researcher	[44]
Cardiovascular complaint over the past month	All	25%	study participants	Interview with psychiatrist	[47]
Chronic health condition	10-12	5%	arrested	Medical examination record	[32]
	13-15	6%	arrested	Medical examination record	[23]
	All	18%	examined	Medical examination record	[42]
	≥ 13	10%	examined	Medical examination record	[27]
	≥ 15	21%	examined	Medical examination record	[26]
	≥ 18	33%	study participants	Questionnaire	[39]
	16-17	7%	examined	Medical examination record	[23]
	Women ≥ 13	23%	examined	Medical examination record	[38]
Chronic infectious disease	Men ≥ 13	1%	examined	Medical examination record	[38]
	Women ≥ 13	1%	examined	Medical examination record	[38]
Chronic somatic disorder	≥ 13	10-12%	examined	Medical examination record	[33]
	≥ 13	18%	examined	Medical examination record	[27]
	< 60	9%	examined	Medical examination record	[25]
	≥ 60	36%	examined	Medical examination record	[25]
Communicable diseases	All	3%	study participants	Interview with psychiatrist	[47]
Conjunctivitis	10-12	2%	arrested	Medical examination record	[32]

Health(care) category	Age (years) and/or gender *	Percentage of study population	Of total detainees	Information source	Publication
COPD (chronic obstructive pulmonary disease)	All	1%	examined	HCP activity questionnaire	[28]
Deep vein thrombosis	All	<1%	examined	Medical examination record	[40]
Deep vein thrombosis / pulmonary embolism / leg ulcer	≥ 16	4%	study participants	Questionnaire	[34]
Diabetes	18-49	3%	arrested	Interview with researcher	[44]
	≥ 50	12%	arrested	Interview with researcher	[44]
	All	2%	examined	Medical examination record	[31]
	All	2%	examined	Medical examination record	[40]
	All	7%	examined	HCP activity questionnaire	[28]
	≥ 13	2%	examined	Medical examination record	[27]
	≥ 16	4%	study participants	Questionnaire	[34]
	≥ 18	5%	study participants	Questionnaire	[39]
	≥ 60	27%	examined	Medical examination record	[24]
	≥ 60	16%	examined	Medical examination record	[25]
	Men ≥ 13	2%	examined	Medical examination record	[38]
	Women ≥ 13	4%	examined	Medical examination record	[38]
	All	5%	study participants	Interview with psychiatrist	[47]
Epilepsy	18-49	2%	arrested	Interview with researcher	[44]
	≥ 50	2%	arrested	Interview with researcher	[44]
	All	4%	examined	Medical examination record	[31]
	All	4%	examined	Medical examination record	[40]
	≥ 13	1%	examined	Medical examination record	[27]
	≥ 16	5%	study participants	Questionnaire	[34]
	≥ 18	4%	study participants	Questionnaire	[39]
	≥ 60	1%	examined	Medical examination record	[24]
	Men ≥ 13	1%	examined	Medical examination record	[38]
	Women ≥ 13	2%	examined	Medical examination record	[38]
	All	2%	study participants	Interview with psychiatrist	[47]
Fatigue	≥ 60	5%	examined	Medical examination record	[24]
Functional limitation: (severe) limitation	10-12	3%	arrested	Medical examination record	[32]
	13-15	2%	arrested	Medical examination record	[23]
	All	8%	examined	Medical examination record	[42]
	≥ 13	1-5%	examined	Medical examination record	[33]
	≥ 13	5%	examined	Medical examination record	[27]
	≥ 18	29%	examined	Questionnaire	[39]
	16-17	2%	examined	Medical examination record	[23]
	< 60	4%	examined	Medical examination record	[25]
	≥ 60	18%	examined	Medical examination record	[25]
	Men ≥ 13	5%	examined	Medical examination record	[38]
	Women ≥ 13	10%	examined	Medical examination record	[38]
Gastro-intestinal symptoms	18-49	17%	arrested	Interview with researcher	[44]
	≥ 50	43%	arrested	Interview with researcher	[44]
	≥ 16	5%	study participants	Questionnaire	[34]
Haemorrhoids	≥ 16	<1%	study participants	Questionnaire	[34]
Head injury	All	6%	examined	Medical examination record	[40]
Heart disease	All	3%	examined	Medical examination record	[40]
	≥ 13	1%	examined	Medical examination record	[27]
	≥ 60	11%	examined	Medical examination record	[25]
	≥ 60	20%	examined	Medical examination record	[24]
	Men ≥ 13	1%	examined	Medical examination record	[38]
	Women ≥ 13	3%	examined	Medical examination record	[38]
Hepatitis B/C	All	<1%	examined	Medical examination record	[40]
	All	2%	examined	HCP activity questionnaire	[28]
	≥ 16	4%	study participants	Questionnaire	[34]

Health(care) category	Age (years) and/or gender *	Percentage of study population	Of total detainees	Information source	Publication
HIV (human immunodeficiency virus)	All	<1%	examined	Medical examination record	[40]
	All	1%	examined	HCP activity questionnaire	[28]
	≥ 16	<1%	study participants	Questionnaire	[34]
Hypertension	18-49	6%	arrested	Interview with researcher	[44]
	≥ 50	18%	arrested	Interview with researcher	[44]
	All	2%	examined	Medical examination record	[40]
	All	7%	examined	HCP activity questionnaire	[28]
	≥ 16	4%	study participants	Questionnaire	[34]
	≥ 60	33%	examined	Medical examination record	[24]
Hypertension, arterial	≥ 13	2%	examined	Medical examination record	[27]
	≥ 18	6%	examined	Medical examination record	[39]
	≥ 60	19%	examined	Medical examination record	[25]
	Men ≥ 13	2%	examined	Medical examination record	[38]
	Women ≥ 13	3%	examined	Medical examination record	[38]
Learning disability	≥ 18	4%	arrested	Interview with researcher	[46]
Musculoskeletal problems	All	4%	examined	HCP activity questionnaire	[28]
	All	7%	examined	Medical examination record	[40]
	≥ 16	4%	study participants	Questionnaire	[34]
Pain	All	7%	examined	Medical examination record	[31]
	≥ 60	32%	examined	Medical examination record	[24]
Physical or mental disorder/handicap	All	30%	arrested	Police custody record	[31]
Polycythemia	≥ 16	<1%	study participants	Questionnaire	[34]
Pregnancy (women)	All	7%	examined	Medical examination record	[40]
	≥ 13	6%	examined	Medical examination record	[38]
	All	3%	study participants	Interview with psychiatrist	[47]
Rheumatic disease	≥ 60	2%	examined	Medical examination record	[24]
Rickets	≥ 16	<1%	study participants	Questionnaire	[34]
Stroke (CVA/TIA)	All	<1%	examined	HCP activity questionnaire	[28]
Thyroid	≥ 16	<1%	study participants	Questionnaire	[34]
(Traumatic) injury	10-12	9%	arrested	Medical examination record	[32]
	13-15	8%	arrested	Medical examination record	[23]
	All	23%	examined	Medical examination record	[40]
	All	27%	examined	Medical examination record	[42]
	≥ 13	20-25%	examined	Medical examination record	[33]
	≥ 13	22%	examined	Medical examination record	[27]
	16-17	10%	examined	Medical examination record	[23]
	< 60	22%	examined	Medical examination record	[25]
	≥ 60	28%	examined	Medical examination record	[25]
	All	15%	study participants	Interview with psychiatrist	[47]
Tuberculosis	≥ 16	1%	study participants	Questionnaire	[34]
**Mental health and psychiatric disorders**					
Mental health problems	All	31%	arrested	Clinical health data exchange	[49]
	All	9%	examined	Questionnaire	[29]
	All	17%	examined	Medical examination record	[31]
	All	38%	examined	HCP activity questionnaire	[28]
	All	50%	examined	Medical examination record	[43]
	≥ 16	11%	study participants	Questionnaire	[34]
	≥ 18	22%	study participants	Questionnaire	[39]
	All	39%	study participants	Interview with psychiatrist	[47]
Mental health problems or depression	All	22%	arrested	Police custody record	[31]
Mental illness (major depression and/or psychotic disorder)	≥ 18	29%	arrested	Interview with researcher	[46]
	All	5%	arrested	Medical examination record	[21]

Health(care) category	Age (years) and/or gender *	Percentage of study population	Of total detainees	Information source	Publication
Mental problems other than alcohol or drug related	< 25	2%	arrested	Medical examination record	[21]
	25-34	5%	arrested	Medical examination record	[21]
	35-44	6%	arrested	Medical examination record	[21]
	≥ 45	6%	arrested	Medical examination record	[21]
	Men, all	4%	arrested	Medical examination record	[21]
	Women, all	8%	arrested	Medical examination record	[21]
Psychiatric disorders / psychopathology	All	22%	arrested	Police custody record	[31]
	All	34%	arrested	Nursing data	[35]
	All	39%	arrested	Interview with researcher	[21]
	13-15	3%	arrested	Medical examination record	[23]
	15-41	11%	arrested	Psychiatric case register	[50]
	< 25	27%	arrested	Interview with researcher	[21]
	25-34	52%	arrested	Interview with researcher	[21]
	35-44	34%	arrested	Interview with researcher	[21]
	≥ 45	48%	arrested	Interview with researcher	[21]
	Men, all	38%	arrested	Interview with researcher	[21]
	Women, all	58%	arrested	Interview with researcher	[21]
	16-17	4%	examined	Medical examination record	[23]
(History of) psychiatric disorders	10-12	7%	arrested	Medical examination record	[32]
	All	37%	examined	HCP activity questionnaire	[37]
	≥ 13	6-9%	examined	Medical examination record	[33]
	≥ 13	6%	examined	Medical examination record	[27]
	< 60	6%	examined	Medical examination record	[25]
	≥ 60	4%	examined	Medical examination record	[24]
	≥ 60	13%	examined	Medical examination record	[25]
	Men ≥ 13	6%	examined	Medical examination record	[38]
	Women ≥ 13	15%	examined	Medical examination record	[38]
Affective disorder	All	5%	arrested	Nursing data	[35]
	All	11%	arrested	Psychiatric case register	[35]
Affective psychoses	15-41	<1%	arrested	Psychiatric case register	[50]
Agitation or aggressiveness	≥ 15	4%	examined	Medical examination record	[26]
Anxiety disorder	All	8%	arrested	Psychiatric case register	[35]
	All	2%	examined	HCP activity questionnaire	[37]
Anxiety without aggressiveness	≥ 15	10%	examined	Medical examination record	[26]
Bipolar disorder	All	<1%	examined	Medical examination record	[40]
	All	1%	examined	HCP activity questionnaire	[37]
	All	3%	examined	HCP activity questionnaire	[28]
Confusion	≥ 15	1%	examined	Medical examination record	[26]
Depressive disorder, moderate	All	5%	study participants	Interview with psychiatrist	[47]
Depressive disorder, severe	All	0%	study participants	Interview with psychiatrist	[47]
Depressive episode (current, major)	≥ 18	22%	arrested	Interview with researcher	[46]
Depressive symptoms	All	11%	examined	HCP activity questionnaire	[37]
	All	13%	examined	Medical examination record	[40]
	All	19%	examined	HCP activity questionnaire	[28]
	≥ 15	1%	examined	Medical examination record	[26]
	≥ 16	12%	study participants	Questionnaire	[34]
Neurotic disorders	15-41	1%	arrested	Psychiatric case register	[50]
Personality disorders	15-41	<1%	arrested	Psychiatric case register	[50]
	≥ 18	21%	arrested	Interview with researcher	[46]
Psychiatric hospitalization	≥ 60	42%	examined	Medical examination record	[25]
Psychological symptoms	≥ 60	6%	examined	Medical examination record	[24]

Health(care) category	Age (years) and/or gender *	Percentage of study population	Of total detainees	Information source	Publication
Psychosis	≥ 18	7%	arrested	Interview with researcher	[46]
	All	2%	examined	HCP activity questionnaire	[28]
	All	5%	examined	HCP activity questionnaire	[37]
(Possible) psychotic symptoms / illness	All	5%	arrested	Psychiatric case register	[35]
	All	6%	arrested	Nursing data	[35]
	≥ 18	7%	arrested	Interview with researcher	[46]
	All	2%	examined	Medical examination record	[40]
	All	8%	study participants	Interview with psychiatrist	[47]
PTSD (post-traumatic stress disorder)	≥ 18	8%	arrested	Interview with researcher	[46]
Schizophrenia	15-41	<1%	arrested	Psychiatric case register	[50]
	All	<1%	examined	Medical examination record	[40]
	All	3%	examined	HCP activity questionnaire	[37]
	All	6%	examined	HCP activity questionnaire	[28]
Self-injury / self-harm (history)	All	20%	arrested	Police custody record	[31]
	All	11%	examined	Medical examination record	[40]
	All	21%	examined	HCP activity questionnaire	[28]
Ongoing suicidal ideation	All	2%	examined	Medical examination record	[40]
	All	11%	study participants	Interview with psychiatrist	[47]
	Men ≥ 13	1%	examined	Medical examination record	[38]
	Women ≥ 13	5%	examined	Medical examination record	[38]
**Addictive behaviors and disorders, general**					
Any drug at time of arrest	17-24	72%	arrested	Urine specimen analysis	[52]
	≥ 25	66%	arrested	Urine specimen analysis	[52]
Intoxicated at time of arrest (drugs or alcohol)	All	18%	examined	HCP activity questionnaire	[37]
Addictive disorder related to psychoactive substance use	≥ 15	71%	examined	Medical examination record	[26]
Substance use (current)	≥ 18	60%	arrested	Interview with researcher	[46]
	All	77%	examined	HCP activity questionnaire	[28]
Substance use disorder / substance abuse	All	28%	arrested	Clinical health data exchange	[49]
	All	70%	arrested	Interview with nurse	[35]
	15-41	3%	arrested	Psychiatric case register	[50]
	All	10%	examined	HCP activity questionnaire	[37]
**Alcohol use**					
Alcohol abuse	All	11%	arrested	Interview with nurse	[35]
	≥ 13	10-12%	examined	Medical examination record	[33]
	≥ 13	29%	examined	Medical examination record	[27]
	≥ 15	32%	examined	Medical examination record	[26]
Alcohol abuse / dependent drinker	All	34%	examined	Medical examination record	[42]
Alcohol consumption	10-12	0%	arrested	Medical examination record	[32]
	13-15	5%	arrested	Medical examination record	[23]
	17-24	22%	arrested	Urine specimen analysis	[52]
	≥ 25	24%	arrested	Urine specimen analysis	[52]
	All	41%	examined	HCP activity questionnaire	[37]
	All	66%	examined	HCP activity questionnaire	[28]
	≥ 16	25%	study participants	Questionnaire	[34]
	16-17	16%	examined	Medical examination record	[23]
Alcohol consumption: > 3 standard units daily, or at least once a week > 5 units	< 25	10%	arrested	Interview with researcher	[21]
	25-34	20%	arrested	Interview with researcher	[21]
	35-44	23%	arrested	Interview with researcher	[21]
	≥ 45	28%	arrested	Interview with researcher	[21]
	All	21%	arrested	Interview with researcher	[21]
	Men, all	20%	arrested	Interview with researcher	[21]

Health(care) category	Age (years) and/or gender *	Percentage of study population	Of total detainees	Information source	Publication
	Women, all	22%	arrested	Interview with researcher	[21]
Alcohol consumption, daily	≥ 13	3-8%	examined	Medical examination record	[33]
	≥ 13	12%	examined	Medical examination record	[27]
	≥ 18	12%	study participants	Questionnaire	[39]
	< 60	12%	examined	Medical examination record	[25]
	≥ 60	35%	examined	Medical examination record	[25]
	Men ≥ 13	8%	examined	Medical examination record	[38]
	Women ≥ 13	5%	examined	Medical examination record	[38]
Alcohol consumption, regular	≥ 60	41%	examined	Medical examination record	[24]
Alcohol consumption level of hazardous or harmful	≥ 18	26%	arrested	Interview with researcher	[46]
Alcohol dependence	≥ 13	2-4%	examined	Medical examination record	[33]
	≥ 13	19%	examined	Medical examination record	[27]
	≥ 16	1%	study participants	Questionnaire	[34]
Alcohol misuse	All	46%	examined	Medical examination record	[31]
Alcohol related medical problems	All	3%	arrested	Medical examination record	[21]
	< 25	1%	arrested	Medical examination record	[21]
	25-34	2%	arrested	Medical examination record	[21]
	35-44	4%	arrested	Medical examination record	[21]
	≥ 45	4%	arrested	Medical examination record	[21]
	Men, all	3%	arrested	Medical examination record	[21]
	Women, all	2%	arrested	Medical examination record	[21]
At risk of alcohol withdrawal	18-49	15%	arrested	Interview with researcher	[44]
	≥ 50	26%	arrested	Interview with researcher	[44]
	All	18%	study participants	Interview with psychiatrist	[47]
Drunk	All	79%	arrested	Police custody record	[31]
**Drug use**					
Drug misuse	All	19%	examined	Medical examination record	[31]
	All	33%	examined	HCP activity questionnaire	[28]
Drug-related medical problems	All	8%	examined	Medical examination record	[21]
	< 25	1%	examined	Medical examination record	[21]
	25-34	6%	examined	Medical examination record	[21]
	35-44	12%	examined	Medical examination record	[21]
	≥ 45	16%	examined	Medical examination record	[21]
	Men, all	8%	examined	Medical examination record	[21]
	Women, all	12%	examined	Medical examination record	[21]
Illicit drug use	10-12	0%	arrested	Medical examination record	[32]
	All	42%	examined	HCP activity questionnaire	[37]
	≥ 13	32%	examined	Medical examination record	[27]
	≥ 60	0%	examined	Medical examination record	[24]
Psychoactive drugs misuse	≥ 15	1%	examined	Medical examination record	[26]
Amphetamines	All	6%	arrested	Interview with nurse	[35]
	17-24	6%	arrested	Urine specimen analysis	[52]
	≥ 25	6%	arrested	Urine specimen analysis	[52]
	All	1%	examined	HCP activity questionnaire	[28]
	All	12%	examined	HCP activity questionnaire	[37]
	≥ 15	<1%	examined	Medical examination record	[26]
Benzodiazepines	All	2%	arrested	Interview with nurse	[35]
	17-24	10%	arrested	Urine specimen analysis	[52]
	≥ 25	20%	arrested	Urine specimen analysis	[52]
	All	1%	examined	HCP activity questionnaire	[28]
	All	4%	examined	HCP activity questionnaire	[37]
	≥ 16	17%	study participants	Questionnaire	[34]
Cannabis consumption	All	6%	arrested	Interview with nurse	[35]

Health(care) category	Age (years) and/or gender *	Percentage of study population	Of total detainees	Information source	Publication
	All	24%	arrested	Interview with researcher	[21]
	≥ 18	23%	arrested	Interview with researcher	[46]
	≥ 25	41%	arrested	Urine specimen analysis	[52]
	≥ 45	18%	arrested	Interview with researcher	[21]
	13-15	12%	arrested	Medical examination record	[23]
	17-24	55%	arrested	Urine specimen analysis	[52]
	< 25	32%	arrested	Interview with researcher	[21]
	25-34	21%	arrested	Interview with researcher	[21]
	35-44	20%	arrested	Interview with researcher	[21]
	Men, all	25%	arrested	Interview with researcher	[21]
	Women, all	10%	arrested	Interview with researcher	[21]
	All	7%	examined	HCP activity questionnaire	[37]
	All	14%	examined	HCP activity questionnaire	[28]
	All	32%	examined	Medical examination record	[42]
	≥ 13	19-37%	examined	Medical examination record	[33]
	≥ 13	29%	examined	Medical examination record	[27]
	≥ 15	35%	examined	Medical examination record	[26]
	≥ 16	21%	study participants	Questionnaire	[34]
	≥ 18	43%	study participants	Questionnaire	[39]
	16-17	35%	examined	Medical examination record	[23]
	≥ 60	1%	examined	Medical examination record	[25]
	Men ≥ 13	30%	examined	Medical examination record	[38]
Cocaine consumption	All	17%	arrested	Interview with researcher	[21]
	13-15	0%	arrested	Medical examination record	[23]
	17-24	21%	arrested	Urine specimen analysis	[52]
	18-49	13%	arrested	Interview with researcher	[44]
	< 25	3%	arrested	Interview with researcher	[21]
	25-34	20%	arrested	Interview	[21]
	≥ 25	30%	arrested	Urine specimen analysis	[52]
	35-44	25%	arrested	Interview	[21]
	≥ 45	30%	arrested	Interview	[21]
	≥ 50	4%	arrested	Interview with researcher	[44]
	Men, all	17%	arrested	Interview with researcher	[21]
	Women, all	25%	arrested	Interview with researcher	[21]
	All	<1%	examined	HCP activity questionnaire	[37]
	All	3%	examined	Medical examination record	[42]
	All	9%	examined	HCP activity questionnaire	[28]
	≥ 13	0-3%	examined	Medical examination record	[33]
	≥ 13	2%	examined	Medical examination record	[27]
	≥ 15	4%	examined	Medical examination record	[26]
	16-17	<1%	examined	Medical examination record	[23]
	< 60	2%	examined	Medical examination record	[25]
	≥ 60	<1%	examined	Medical examination record	[25]
	Women ≥ 13	4%	examined	Medical examination record	[38]
	Men ≥ 13	1%	examined	Medical examination record	[38]
Crack consumption	≥ 18	7%	arrested	Interview with researcher	[46]
	All	10%	examined	HCP activity questionnaire	[28]
	≥ 16	34%	study participants	Questionnaire	[34]
	≥ 18	8%	study participants	Questionnaire	[39]
	13-17	0%	examined	Medical examination record	[23]
	All	22%	study participants	Interview with psychiatrist	[47]
Heroin consumption	All	6%	arrested	Interview with nurse	[35]
	≥ 18	4%	arrested	Interview with researcher	[46]
	All	1%	examined	Medical examination record	[42]
	≥ 13	0-1%	examined	Medical examination record	[33]
	≥ 13	1%	examined	Medical examination record	[27]
	≥ 16	34%	study participants	Questionnaire	[34]

Health(care) category	Age (years) and/or gender *	Percentage of study population	Of total detainees	Information source	Publication
	≥ 18	2%	study participants	Questionnaire	[39]
	13-17	<1%	examined	Medical examination record	[23]
	< 60	2%	examined	Medical examination record	[25]
	≥ 60	<1%	examined	Medical examination record	[25]
	Women ≥ 13	1%	examined	Medical examination record	[38]
	Men ≥ 13	1%	examined	Medical examination record	[38]
	All	11%	study participants	Interview with psychiatrist	[47]
LSD	≥ 15	<1%	examined	Medical examination record	[26]
Opiates	All	9%	arrested	Interview with researcher	[21]
	≥ 25	38%	arrested	Urine specimen analysis	[52]
	< 25	1%	arrested	Interview with researcher	[21]
	17-24	27%	arrested	Urine specimen analysis	[52]
	18-49	9%	arrested	Interview with researcher	[44]
	25-34	7%	arrested	Interview with researcher	[21]
	35-44	18%	arrested	Interview with researcher	[21]
	≥ 45	16%	arrested	Interview with researcher	[21]
	≥ 50	7%	arrested	Interview with researcher	[44]
	Men, all	9%	arrested	Interview with researcher	[21]
	Women, all	16%	arrested	Interview with researcher	[21]
	All	1%	examined	HCP activity questionnaire	[28]
	All	20%	examined	HCP activity questionnaire	[37]
	≥ 15	5%	examined	Medical examination record	[26]
**Opinion on overall health rating**					
(Very) good opinion on overall health	10-12	85%	arrested	Medical examination record	[32]
	13-15	88%	arrested	Medical examination record	[23]
	All	84%	examined	Medical examination record	[42]
	≥ 13	77-88%	examined	Medical examination record	[33]
	16-17	84%	examined	Medical examination record	[23]
	< 60	89%	examined	Medical examination record	[25]
	≥ 60	60%	examined	Medical examination record	[25]
	Men ≥ 13	89%	examined	Medical examination record	[38]
	Women ≥ 13	70%	examined	Medical examination record	[38]
Deteriorated perceived health (average, bad or very bad)	≥ 18	23%	study participants	Questionnaire	[39]
**Health concerns during custody**					
Health concern in custody, any	All	22%	arrested	Questionnaire	[51]
Access to healthcare as major concern	≥ 18	11%	study participants	Interview with researcher	[48]
Alcohol problem concern whilst in custody	All	4%	arrested	Questionnaire	[51]
Drug problem concern whilst in custody	All	6%	arrested	Questionnaire	[51]
Medication concern whilst in custody	All	8%	arrested	Questionnaire	[51]
Mental health problem concern whilst in custody	All	2%	arrested	Questionnaire	[51]
Self-harm or suicide concern whilst in custody	All	<1%	arrested	Questionnaire	[51]
	≥ 18	18%	arrested	Interview with researcher	[46]
Suicidal thoughts / depression / stress as major concern	≥ 18	3%	study participants	Interview with researcher	[48]

**Health(care) category**	**Age (years) and/or gender ***	**Percentage of study population**	**Of total detainees**	**Information source**	**Publication**
Traumatic injury concern whilst in custody	All	2%	arrested	Questionnaire	[51]
**Intellectual disability**					
Intellectual disability	All	3%	study participants	Interview with psychiatrist	[47]
Possible pseudo-dementia	All	<1%	study participants	Interview with psychiatrist	[47]
**Treatment prior to custody**					
Treatment prior to custody	All<br>≥ 15<br>≥ 60	9%<br>24%<br>72%	examined<br>examined<br>examined	Medical examination record<br>Medical examination record<br>Medical examination record	[42]<br>[26]<br>[24]
Addiction treatment, current or past	≥ 13<br>≥ 13	1-2%<br>1-5%	examined<br>examined	Medical examination record<br>Medical examination record	[33]<br>[33]
Opioid substitution therapy	≥ 18	7%	study participants	Questionnaire	[39]
**Chronic medical treatment**	≥ 13	16-23%	examined	Medical examination record	[33]
Previous heart attack	All	1%	examined	HCP activity questionnaire	[28]
**Mental health disorder treatment**	≥ 18	13%	study participants	Questionnaire	[39]
Contacted by public mental health team within previous 5 years	All<br>< 25<br>≥ 45	26%<br>10%<br>37%	examined<br>examined<br>examined	Outpatient public mental health services register<br>Outpatient public mental health services register<br>Outpatient public mental health services register	[21]<br>[21]<br>[21]
Psychiatric disorder care	All<br>Men ≥ 13<br>Women ≥ 13	32%<br>3%<br>7%	arrested<br>examined<br>examined	Psychiatric case register<br>Medical examination record<br>Medical examination record	[35]<br>[38]<br>[38]
**Medication**, any	18-49<br>≥ 50<br>All<br>All	36%<br>67%<br>54%<br>41%	arrested<br>arrested<br>examined<br>study participants	Interview with researcher<br>Interview with researcher<br>HCP activity questionnaire<br>Interview with psychiatrist	[44]<br>[44]<br>[28]<br>[47]
Prescribed medication	All	29%	examined	Medical examination record	[31]
(Supposed to be) taking tablets/medications	All	20%	arrested	Police custody record	[31]
Antidepressant medication	All<br>All	12%<br>7%	arrested<br>study participants	Psychiatric case register<br>Interview with psychiatrist	[35]<br>[47]
Antipsychotic medication	All<br>All	2%<br>3%	arrested<br>study participants	Psychiatric case register<br>Interview with psychiatrist	[35]<br>[47]
Benzodiazepine prescription	All	12%	arrested	Psychiatric case register	[35]
Oral contraceptives	Women ≥ 13	7%	examined	Medical examination record	[38]
Psychoactive treatment prior to custody	≥ 13<br>≥ 18	6-12%<br>15%	examined<br>study participants	Medical examination record<br>Questionnaire	[33]<br>[39]
Psychotropic medication	≥ 18	17%	arrested	Interview with researcher	[46]

* (Sub)groups other than age and gender not reported in this table.
