# Supplementary material for: Defining quality of healthcare in Dutch police custody: the development of a conceptual framework for monitoring care quality through a scoping review and expert consultations
Source: BMC Public Health. 2026 Jul 2;26:2128. doi: 10.1186/s12889-026-27949-2 (PMC13359842; doi:10.1186/s12889-026-27949-2)
Supplement: Supplementary file 5 — Supplementary Material 5. [file 12889_2026_27949_MOESM5_ESM.pdf]

Supplementary Table S4: Recommendations to ensure or improve quality of care in short-term police custody settings

Domain	Subdomain	Suggested quality of care aspect	Publication
Access to care	Medical assessment	Police officers should even seek medical attention for persons who seem 'only inebriated'.	[57]
Access to care	Medical assessment	If medical examination is required due to doubts on the person's fitness for custody, police officers must not let themselves be sent away by paramedics or nurses.	[60]
Access to care	Medical assessment	Opportunities for improvement in detainee outcomes pertained to the inadequate assessment/identification of physical and mental health issues.	[19]
Access to care	Medical assessment	A police (custody) officer should call in medical assistance when an apparently intoxicated person causes problems in the public domain or when brought to a police station.	[58]
Access to care	Medical assessment	Prevent neglectful punitive practices, which possibly also procedures and exacerbates physical and psychological harms, including injury from violence and self-harm, and painful and distressing withdrawal symptoms.	[63]
Access to care	Referral	Persons with more than minor head injuries should, if in doubt, always receive CT or X-ray examination.	[57]
Access to care	Timeliness	Opportunities for improvement in detainee outcomes pertained the length of time to access health care.	[19]
Access to care	Timeliness	It has to be ensured that policemen are able to consult a doctor quickly even at night or on weekends.	[62]
Access to care	Timeliness	Medical attention should be given as soon as possible.	[61]
Access to care	Timeliness	All calls should be responded to within a 2-hour period.	[51]
Access to care	Timeliness	Attention should be paid to the period of apprehension and transport by the police, i.e. before entering the police station, as it appears to be a high-risk setting for the occurrence of fatal medical emergencies.	[58]
Continuity of information	Availability of information	Information on the outcomes of hospital referral during custody should also be sent to the (referring) forensic physician, in addition to only the general practitioner of the patient.	[36]
Continuity of information	Availability of information	A patient transfer form between police custody and the hospital with: referring information, a patient signed consent section and space for return relevant medical information can improve communication and information transfer between the police custody (healthcare staff) and the hospital.	[45]
Continuity of information	Availability of information	Further collaboration and information sharing between the custody healthcare team and the community and hospital based services is needed to be able to provide a more consistent patient care.	[68]
Continuity of information	Availability of information	Closer partnerships between the police and acute mental health teams are required to understand the complex needs an offender may exhibit and can improve situations where police staff has to work with limited background knowledge of cases having little access to medical records.	[67]
Continuity of information	Availability of information	Continuity of care between mental illness and the criminal justice system include timely contact with treating clinicians, and the willingness of unwell detainees to inform healthcare professionals in custody of their conditions and consent to receiving medication in police cells.	[35]
Continuity of information	Availability of information	It should be noted that Liaison and Diversion clinicians can face challenges of interagency working, cultural barriers between providers, imperfect information technology (especially when working between remote sites), technology systems in different providers which do not 'talk' to each other, and agencies who are reticent to share information citing fear of flouting data protection regulations.	[69]

Continuity of information	Availability of information	It's recommended that the institutions involved in the penal medical systems (i.e. the correctional institutions, mental health care, police and forensic medical practitioners) make provisional agreements about the transfer of medical data, until there is complete interconnection of the various electronic file systems. Optimalization of medical data transfer from and to prison is of great importance in the continuity of care.	[66]
Continuity of information	Availability of information	Highlight medical condition (diabetes) on police database for future reference.	[65]
Continuity of information	Communication	The examining physician should be fully informed about any details of the case history especially in respect to any observed intake of a large number of tablets or binge drinking.	[57]
Continuity of information	Communication	The doctor should prescribe the mode of monitoring including intervals and procedures (e.g. checking if a person can be woken) or should impose concrete conditions such as requirements on the place of detention, food intake and administration of medication.	[57]
Continuity of information	Communication	The doctor should be required to state his/her findings and potential instructions legibly and in a manner comprehensible to non-professionals.	[57]
Continuity of information	Communication	Officers should be obliged to countersign the certificate as confirmation that they have been informed and understand the result of the medical assessment and any conditions imposed.	[57]
Continuity of information	Communication	A written handover should always be supported with a verbal handover to the custody sergeant.	[67]
Continuity of information	Communication	A lack of information caused by the absence of a thorough handover of findings, medication policies, and any prior history of suspects assessed by a mental health crisis service can adversely affect the care provided to detainees by forensic psychiatrists and doctors, as well as the quality of the preliminary examination (examination in order to advice which care a detainee need within the justice system).	[70]
Coordination of care	Collaboration	Regular interactions and communication facilitates collaboration between custody nurses and desk sergeants, in particular by understanding each other's tasks and practices, a shared goal and respect for each other's skills.	[80]
Coordination of care	Collaboration	Treatment services for illicit drug-dependent arrestees not currently receiving treatment should be made available to address unmet treatment needs.	[52]
Coordination of care	Collaboration	Referring arrested individuals for drug use to community-based treatment centers by the police and the Centers for Disease Control could potentially reduce criminal behavior.	[72]
Coordination of care	Collaboration	The medical examination in custody could act as an opportunity for adolescents to have access to health care, beyond their immediate health needs in detention, possibly associated with brief interventions in the field of addictive behaviors.	[23]
Coordination of care	Collaboration	Arrested drug drivers are generally young, healthy, and infrequently reported assaults or presented traumatic injuries, which does not put them in a high risk for medical conditions. Yet, medical care in custody of detainees arrested in relation to substance misuse could include brief interventions on addictive behaviors.	[79]
Coordination of care	Collaboration	Interventions to increase GP registration after contact with mental health services in police custody (especially for homeless detainees) can improve onward referral for acute needs.	[76]
Coordination of care	Collaboration	Understanding the mental health and substance use disorder diagnoses among arrestees may be an opportunity to divert individuals to treatment or to assign other resources within the justice system. Linked clinical and police data are essential for the early detection.	[49]

Coordination of care	Collaboration	Health promotion and screening during police custody detention requires further development and could be integrated into routine health care services. However, the efficacy of current police administered health screening procedures, only picks up a proportion of medical problems and an even smaller proportion of mental health problems.	[68]
Coordination of care	Collaboration	Collaborative working for individuals under the Mental Health Act (e.g. basing a community psychiatric nurse in the police control room, streamlining processes for all officers, introduction of a single contact number for a coordinator) can improve the relations between the police and health frontline workers and care benefit those individuals.	[74]
Coordination of care	Collaboration	Police, justice, mental healthcare services, and addiction services would do well to develop further policies around the specific issues of sobering up (e.g. in terms of response times).	[78]
Coordination of care	Collaboration	The level of engagement by the police with those who are mentally ill may continue to rise in the absence of access to alternative support for this in mental health crisis.	[75]
Coordination of care	Collaboration	It should be clearly understood by hospital medical staff that the routine care and observations of detainees in custody is by civilian custody staff and that the role of the forensic physician/nurse is primarily assessment and advice. Therefore, patients should not be sent back to custody until fit for police and civilian staff routine cell checks type care.	[45]
Coordination of care	Collaboration	It is important to understand the type and range of unmet health and social needs in detainees presenting with mental illness in police custody. To improve the safety and well-being of these detainees, consistent health arrangements that work throughout the criminal justice pathway should be commissioned, together with more standardized forms of collaboration between the police and healthcare professionals.	[46]
Coordination of care	Collaboration	Closer partnerships between the emergency departments and ambulance trusts are required to manage the complex needs of some offenders, especially where there is minimal facilities for healthcare on site in the police custody setting.	[67]
Coordination of care	Collaboration	Newly arrested youth should receive a full range of screening for physical and mental health issues (including biomarkers of health status to the extent possible) and an indicated treatment follow-up. An intake facility can serve as critical community public health and behavioral health resources, to identify and respond to the services needs of arrested youth, whose families often lack the resources to access these services on their own.	[73]
Coordination of care	Collaboration	Introducing universal mental health screening for all detainees needs to be accompanied by appropriate, responsive and proactive clinical services working hand-in-hand with well-trained police officers to facilitate access to treatments which match people's needs.	[71]
Coordination of care	Collaboration	Medical examination in custody could be an opportunity to restore psychiatric care for arrestees with interrupted mental health care among those reporting mental disorders.	[29]
Coordination of care	Coordination of transition	Transport movements of detainees from a temporary holding cell back and forth to an overnight cell carry risks, such as the possibility of leaving medication behind.	[64]
Coordination of care	Coordination of transition	(Forensic) physicians from police custody settings should communicate and share medication information with general practitioners and prison medical officers.	[77]
Coordination of care	Coordination of transition	Factors affecting liaison and diversion services are: frequent structural changes, both internal (e.g. frequent staff changes, such as detention officer turnover) or external, such as centrally driven transformation (e.g. splitting of probation).	[69]

Detection of care needs	Detection of care needs of detainee	When using early warning score systems to triage police custody detainees, such systems should support clinical judgement, not drive it. Training is required if non-medical police staff are to be entrusted with the responsibility of assigning a score.	[100]
Detection of care needs	Detection of care needs of detainee	Clear medical guidance should be established for clinical conditions for which medical assessment is always required (e.g. head injuries, obvious signs of intoxication, alcohol withdrawal, mental health).	[83]
Detection of care needs	Monitoring	The police, even if over-stretched, should adhere to the monitoring schedule.	[57]
Detection of care needs	Monitoring	Monitoring and responsibility for unconscious or unresponsive persons (e.g. persons with an alcohol or drug-addiction syndrome) cannot be assumed by the police alone.	[57]
Detection of care needs	Monitoring	Police personnel should not hesitate to call medical assistance again if there is a significant change in the detainee's condition such as an increasing dullness of consciousness in cerebrocranial trauma or an onset of withdrawal symptoms.	[57]
Detection of care needs	Monitoring	Undertake intensified supervision and counselling of high-risk individuals for self-harm or suicidal behavior.	[56]
Detection of care needs	Monitoring	As high rates of drug and alcohol misuse and chronic health conditions are present in detainees, screening and assessment in short-term custodial settings is essential not only on arrival, but throughout the detention period.	[19]
Detection of care needs	Monitoring	The doctor should specify in writing, on the medical certificate, the details of the medical surveillance required for the detainee to remain in custody.	[61]
Effectiveness	Health outcomes	Opportunities for improvement in detainee outcomes pertained the impact on the detainee mental health of short-term detention.	[19]
Effectiveness	Health outcomes	The ability of the police to draw on a range of reliable data sources in addition to detainees' self-report would be an advantage in fulfilling the duty of care requirements regarding health and well-being of detainees in police custody.	[103]
Healthcare provision	Alcohol withdrawal	Special attention should be paid to drink-drivers who refuse or are not able to complete breath alcohol measurement.	[105]
Healthcare provision	Alcohol withdrawal	Adequate medical care and surveillance are needed for daily alcohol drinkers over 60 in police cells, as sudden withdrawal of alcohol consumption can induce severe health complications such as seizures or delirium tremens.	[25]
Healthcare provision	Care for urgent needs	Medical intervention during custody should include the continuity of care regarding any ongoing treatment or medications. This may mean contacting the patient's doctor or family, as elderly arrestees commonly do not have their medication with them.	[24]
Healthcare provision	Care for urgent needs	Detainees with diabetes who required insulin therapy were usually examined three times every 24 hour and had blood glucose monitoring, insulin therapy, and adequate meals.	[24]
Healthcare provision	Drugs withdrawal	Police should exercise increased vigilance when confronted with an (apparently) intoxicated person, particularly if stimulants such as cocaine or alcohol have been consumed. Police (custody) officers should be aware that intoxication can impair a person's ability to coherently indicate, or indicate at all, whether there might be medical problems. They should also recognize that intoxication can overshadow medical problems, and medical conditions can be mistaken for intoxication due to alcohol consumption.	[58]
Healthcare provision	Drugs withdrawal	If methadone was prescribed before detention (e.g. started by an addiction care or psychiatric hospital), the same methadone dose should be continued.	[66]

Healthcare provision	Drugs withdrawal	Problematic consumption of illicit drugs and psychoactive substances are existent among some police custody detainees and signs of withdrawal can be observed.	[41]
Healthcare provision	Medication	Police officers who apprehend suspects in their home should pick up any necessary prescriptions and medication.	[61]
Healthcare provision	Medication	If medication is not available, it could be acceptable for custody officers to go to the pharmacy with a prescription made out by the doctor called upon and deliver the medicines directly to the detainee, if this is done in the detainee's interests and with their consent. If medication is not available and there is no way of paying the pharmacist for prescribed medicines, the use of hospital services is the only solution.	[61]
Healthcare provision	Mental / psychiatric health issues	Detainees with preexisting vulnerabilities should receive higher priority from healthcare workers attending police cells. For instance, effectively managing the high levels of psychopathology should involve targeting individuals with a current psychiatric disorder and those with a history of psychiatric hospitalization	[48]
Healthcare provision	Minors	When the detainee is a child, the clinical examination should include a medical history focusing on diseases that cause problems in custody, mainly including asthma, epilepsy, diabetes, contagious diseases and mental disorders. Detainees 10-12 years of age may have difficulties recalling their medical history and there is a risk of concealed disease.	[32]
Healthcare provision	Older individuals	In cases were physicians consider older detainees unfit for custody even though the detainee's health condition does not require hospital care, the judicial authority or the police officer has the responsibility of finding suitable accommodation or releasing the detainee from custody	[25]
Healthcare provision	Older individuals	Police detainees over 50 should be considered to have a health assessment as routine procedure	[44]
Healthcare provision	Pregnancy	Before the eighth month of pregnancy, asymptomatic arrestees can be considered fit for detention during daytime, depending on material conditions in police cells.	[38]
Healthcare provision	Pregnancy	Careful management of pregnant detainees who use opiates needs to be considered, as miscarriage may be associate with opiate withdrawal.	[67]
Healthcare provision	Pregnancy	Eight-month pregnancy is a contraindication to detention in police stations, even when the clinical examination is normal, as the woman could deliver at any time.	[38]
Healthcare provision	Pregnancy	An initial medical screen at intake presents a public health opportunity for women who have had recent unprotected sex and who want to avoid pregnancy. By addressing reproductive health needs, emergency contraception to incarcerated women could help reduce health disparities.	[107]
Infrastructure and (medical) resources	Cell provisions	Police cells can be a challenging environment for handling physiological conditions such as menstruation and pregnancy, sanitary towels should be available for female arrestees in police cells.	[38]
Infrastructure and (medical) resources	Cell provisions	Beds should have properties that do not carry the risk of fatal falls.	[57]
Infrastructure and (medical) resources	Cell provisions	Restrict access to items useful to inflict self-harm, especially the one by hanging will help to reduce custody-related mortalities.	[56]
Infrastructure and (medical) resources	Cell provisions	The medical examination should take place in the police station, so that the doctor can assess the conditions in which the detainee is being held.	[25]
Infrastructure and (medical) resources	Cell provisions	If the detainee does not have a health problem but the doctor considers the custody conditions to be disgraceful, the doctor may refuse to make any statement on the detainee's fitness for detention.	[61]

Infrastructure and (medical) resources	Cell provisions	If the detainee has a health problem that is incompatible with detention in the police station because of the physical conditions in that police station, the medical certificate of fitness for detention should be issued on condition that certain improvements be made or that the detainee be transferred to facilities where such improvements can be made.	[61]
Infrastructure and (medical) resources	Cell provisions	The detainee should not be restrained in any way when the examination is performed, except in exceptional circumstances.	[61]
Infrastructure and (medical) resources	Clinical diet	All diabetics must be given food prior to insulin administration to avoid hypoglycaemic episodes.	[67]
Infrastructure and (medical) resources	Clinical diet	Proposed and implemented diabetes management equipment and initiatives: a dietetic review by a specialist dietitian regarding te availability of carbohydrate in meals.	[65]
Infrastructure and (medical) resources	(Medical) equipment	Proposed and implemented diabetes management equipment and initiatives: provision of new finger-pricking devices and new blood glucose monitoring equipment subject to external quality assurance.	[65]
Infrastructure and (medical) resources	(Medical) equipment	Proposed and implemented diabetes management equipment and initiatives: a basic stock of different insulins and injection devices should be kept on site.	[65]
Infrastructure and (medical) resources	(Medical) equipment	The facilities made available to the doctor should prove adequate for carrying out a medical examination, but will not be appropriate for advanced medical care such as aseptic suturing or inspection of body cavities.	[61]
Infrastructure and (medical) resources	Interpreter	The interview should be conducted in a language and words that both can understand. An interpreter should be available when the doctor arrives.	[61]
Legal	Medical confidentiality	A clear distinction between templates helps doctors to identify which information they must disclose to the police officers and which information must be kept confidential.	[77]
Legal	Medical confidentiality	The examination should be performed where it cannot be seen or overheard by any third party to preserve the detainee's dignity and the doctor's duty of confidentiality.	[61]
Legal	Medical confidentiality	The doctor's opinion should take the form of a two-part national document. The first part should be a standard medical certificate to be sent to the authority who requested the doctor's attendance. Three copies should be made: one for the requesting authority, one for the doctor, and one for the detainee. The second part, which is not sent to the requesting authority, is the confidential medical record. Two copies should be made: one should be kept by the doctor, the other two may be sent, in a sealed envelope, to the detainee at the end of detention.	[61]
Legal	Medical confidentiality	If detainees have their own supply of medication or if it can be brought by their family, it is recommended that the doctor split up the pack into individually sealed envelopes marked with the detainee’s name and time of administration. In this way, the custody officers can deliver medication and comply with the duty of confidentiality.	[61]
Quality systems	Medication safety	All medications must be checked to confirm current prescription, all details of medication should be entered into the police record system, and all controlled drugs should be required to have a doctor supervise the administration.	[67]
Quality systems	Medication safety	All insulin administration should be supervised by an health care provider and must only be given following a blood glucose check and a meal	[67]
Quality systems	Medication safety	Medication brought from home should be checked by whether: it is not expired, in the detainee’s name, and that it contains correct dispending information for the custody officer.	[67]

Quality systems	Medication safety	A system should be in place to ensure that routine medication for diabetes is not omitted, and that rapid-acting carbohydrate are available at all times.	[65]
Quality systems	Multidisciplinary agreements	Police authority should not design healthcare policies and procedures without reference to healthcare expertise.	[31]
Quality systems	Performance reviews	A forcewide log of all critical incidents in custody (including hospital transfers) that is reviewed on structural basis during custody safety meetings (by custody inspectors, manager of civilian custody staff and medical staff) allows for regular discussion and feedback to all staff on learning points in relation to medical emergencies.	[45]
Quality systems	Performance reviews	Any provider of healthcare in custody should have clearly stated measurable quality standards with regular performance reviews and a focus on continuous quality improvement to ensure that the needs of detainees are being met efficiently and to a high standard.	[31]
Quality systems	Performance reviews	A standardized procedure for handling complaints about healthcare providers should be introduced across all police custodial and forensic healthcare services. Complaints are a valuable source of qualitative information about healthcare service failures, highlighting areas needing improvement and can trigger investigations to review incidents and initiate changes to prevent recurrence. A periodic systematic reviews of all complaints can enhance patient safety and support quality assurance and continuous improvement in clinical practice.	[110]
Quality systems	Performance reviews	Police and Crime Commissioners who are responsible for procuring forensic medical examination services should understand the implications of complaints against healthcare providers and should be aware of the nature and frequency within their own service, so that they can make informed decisions when identifying service providers.	[110]
Quality systems	Performance reviews	An organization for the factual and objective investigation of deaths in police custody should be set up in all European countries and all deaths in police custody should be subject to a post-mortem examination. A systematic analysis of such cases can generate specific prevention measures to reduce the risk of severe health consequences and deaths in police custody in the future. The data should be transparent and openly published in order to permit comparison between countries and institutions to improve universal learning and practice.	[83]
Quality systems	Registration	The development of a complaints database for healthcare providers working in police custodial and forensic medical/healthcare services should be considered.	[110]
Quality systems	Registration	A uniform and complete registration database should be developed for deaths occurring under the responsibility of police forces, which would enable the comparison of data from different countries.	[58]
Satisfaction	Satisfaction and complaints	The attitude and treatment of detainees by custody officers are essential. Creating a calm atmosphere can positively influence the mood of the detainees, which, in turn, can have a favorable effect on the working environment and safety of the custody officers.	[64]
Scope and nature of the care demand	Availability of information about the care demand	Issues concerning the precise needs of special groups, women, young people, ethnic minority groups in particular, need further consideration and investigation.	[103]
Staff	Education and training	Processes and training need to be in place to support detention, escort and custody support officers in carrying out continuous assessment for risks and report any concerns they have.	[60]

Staff	Education and training	Police custody officers are in need of training for completing health risk assessments when detainees are booked in.	[31]
Staff	Education and training	Relevant qualifications should be mandatory for healthcare professionals working in police custody.	[83]
Staff	Education and training	Police should be trained to use telephone triage to assist custody staff in making appropriate decisions and selection by the police of the appropriate venue for detainees to be seen (i.e. A&E).	[67]
Staff	Education and training	Police officers should be trained in the assessment of the state of consciousness of a sleeping person.	[57]
Staff	Education and training	Special attention needs to be given to train police staff to identify suicidal behaviors among individuals in custody. They should be made aware of the importance of seeking the help of a medical professional/psychiatrist at an early stage for individuals at a risk for suicide.	[56]
Staff	Education and training	The large majority of the custody staff expressed that they would like to receive additional training on mental health.	[81]
Staff	Education and training	Training in mental health issues for police officers is key.	[71]
Staff	Education and training	A comprehensive mental health training for custody staff is important.	[84]
Staff	Education and training	Train (healthcare) staff regarding mental health issues, including Section 136 (Mental Health Act) assessments.	[67]
Staff	Education and training	It is recommended that the staff responsible for detainees should receive ongoing training in first aid.	[61]
Staff	Education and training	Police custody officers are in need of training for administering medication.	[31]
Staff	Education and training	Initiatives implemented for diabetes management: display posters on diabetes management and emergency identification for custody suite staff, forensic nurse induction day, regular assessment of staff competencies and provision of updates on diabetes, link nurse to liaise with secondary care team, link tutor to maintain competencies and cascade to peripheral custody units.	[65]
Staff	Skill-mix and capacity	Police officers need the support of mental health professionals in the police station itself.	[59]
Staff	Skill-mix and capacity	Having a standardized access to a dedicated nurse (custody nurse or custody psychiatric nurse) in each custody area would be beneficial for the police custody staff to provide more accurate risk assessments and relevant care provision for detainees. They can also help in cases where the detainee is not necessarily in need of urgent medical support, but still requires a more thorough and ongoing review to assess other social and health needs.	[82]
Staff	Skill-mix and capacity	Having a mental health nurse working in custody would be beneficial, enabling the early identification of mental health concerns, having enough knowledge to deal with the detainee's needs, and manage the levels of risk and vulnerability within the custody environment.	[60]
Staff	Skill-mix and capacity	One good solution is provided by central facilities specializing in police custody which would mean that police officers working there would not be involved in other duties and could therefore concentrate on the necessary checks.	[57]

Staff	Skill-mix and capacity	Appropriate training can lead to greater skill-mix in custody so tasks currently undertaken by forensic medical examiners (FME) can be conducted by other healthcare staff. Advantages: help to address problems of delays experienced by waiting for an FME to arrive, reduce stress and anxiety for detention staff, more efficient medication management, removing the need for detention staff to administer medication, healthcare staff being able to provide advice to detention staff, and the potential to improve after-care in linking detainees with appropriate community services (ensure general practitioner registration and ongoing care).	[31]

* Factors related to clinical near misses described by Webb et al. [67] are rephrased into recommendations to fit this table
